# Elastic deformations mediate interaction of the raft boundary with membrane inclusions leading to their effective lateral sorting

**DOI:** 10.1038/s41598-020-61110-2

**Published:** 2020-03-05

**Authors:** Konstantin V. Pinigin, Oleg V. Kondrashov, Irene Jiménez-Munguía, Veronika V. Alexandrova, Oleg V. Batishchev, Timur R. Galimzyanov, Sergey A. Akimov

**Affiliations:** 1 A.N. Frumkin Institute of Physical Chemistry and Electrochemistry, Russian Academy of Sciences, 31/4 Leninskiy prospekt, Moscow, 119071 Russia; 2 National University of Science and Technology “MISiS”, 4 Leninskiy prospect, Moscow, 119049 Russia; 3 Moscow State University, 1 Leninskie Gory, Moscow, 119991 Russia

**Keywords:** Membrane biophysics, Biological physics

## Abstract

Liquid-ordered lipid domains represent a lateral inhomogeneity in cellular membranes. These domains have elastic and physicochemical properties different from those of the surrounding membrane. In particular, their thickness exceeds that of the disordered membrane. Thus, elastic deformations arise at the domain boundary in order to compensate for the thickness mismatch. In equilibrium, the deformations lead to an incomplete register of monolayer ordered domains: the elastic energy is minimal if domains in opposing monolayers lie on the top of each other, and their boundaries are laterally shifted by about 3 nm. This configuration introduces a region, composed of one ordered and one disordered monolayers, with an intermediate bilayer thickness. Besides, a jump in a local monolayer curvature takes place in this intermediate region, concentrating here most of the elastic stress. This region can participate in a lateral sorting of membrane inclusions by offering them an optimal bilayer thickness and local curvature conditions. In the present study, we consider the sorting of deformable lipid inclusions, undeformable peripheral and deeply incorporated peptide inclusions, and undeformable transmembrane inclusions of different molecular geometry. With rare exceptions, all types of inclusions have an affinity to the ordered domain boundary as compared to the bulk phases. The optimal lateral distribution of inclusions allows relaxing the elastic stress at the boundary of domains.

## Introduction

Cellular membranes are laterally heterogeneous^1–3^. Many membrane proteins are believed to function properly only inside lipid-protein domains, also referred to as rafts^4–7^. Proteins in these domains are surrounded by a more or less thick lipid shell^8–11^. In model purely lipidic systems it is demonstrated that lipids can form similar domains, in which they are in a liquid-ordered (L_o_) phase state, while the surrounding membrane is liquid-disordered (L_d_)^12–15^. Ordered domains are usually bilayer, i.e. exist in both membrane leaflets at the same lateral position^14–17^. Such transbilayer coupling could be driven by elastic deformations arising at the domain boundary and by membrane thermal fluctuations^18–21^. The mechanism of this coupling is not specific to the exact lipid composition of the domain: only the higher ordering of lipids with respect to the surrounding membrane is important^22–24^. A bilayer structure of rafts is believed to be a key property in providing raft-dependent signal transduction across the plasma membrane^4,5^. There are increasing evidences that a raft interior itself may be laterally inhomogeneous: some molecules may prefer its boundary region rather than its bulk part. In particular, the fusion peptide of the human immunodeficiency virus (HIV) gp41 protein functions with the highest efficiency only in the presence of the L_o_/L_d_ phase boundary in the target membrane^25,26^. In addition, the HIV receptor CCR5 preferentially localizes at the domain boundary^26^. It means that the domain boundary may provide an optimal environment necessary for proteins to carry out their biological functions. Besides, so-called line-active membrane components (linactants) are assumed to accumulate at the domain boundary, leading to a sharp decrease of the boundary energy by analogy with surface-active compounds in 3D systems^27,28^. To explain the mechanism of the line activity, a concept of so-called hybrid lipids was proposed^28^: in order to be line-active, hybrid lipids should combine chemical groups preferring L_o_ and L_d_ phases in a single molecule. As an example of a hybrid lipid, palmitoyl-oleoyl-phosphatidylcholine (POPC) is considered^27^. One acyl chain of this lipid is saturated (palmitoyl-) and prefers the L_o_ phase. Another chain is unsaturated (oleoyl-) and should prefer the disordered L_d_ environment. Thus, this lipid is expected to partition to the L_o_/L_d_ phase boundary and to act as a linactant. However, the line activity of POPC has not been observed^29,30^. Therefore, the hybrid nature of the molecule is not a prerequisite for the line activity.

Recently, we have explained the molecular mechanism of the line activity, basing on a thickness mismatch concept^31^. From experiments *in vitro*^31–33^ and *in silico*^24,34,35^ we know that ordered domains are thicker than the surrounding liquid-disordered membrane. If L_o_ and L_d_ phases were homogeneous up to their boundary, a step in the bilayer thickness would exist. This step would be energetically unfavorable, due to a contact between a hydrophobic membrane core and either water or polar lipid heads. The energy of the contact might be decreased at the expense of membrane deformations in the vicinity of the boundary, leading to the reduction, down to zero, of the step amplitude^36^. The elastic energy stored in membrane deformations is minimal when the L_o_ domains in opposing monolayers are not exactly in register, but their boundaries are laterally shifted by 2–4 nm with respect to each other^20,37–39^. Thus, the contact of two bilayer L_o_ and L_d_ phases occurs across the intermediate region of 2–4 nm width, where one monolayer is in the L_o_ state and the other monolayer is in the L_d_ state. Such an equilibrium shift of the domain boundaries is observed in molecular dynamics simulations^24,34,35^. The structure of deformations in this intermediate region favors the accumulation of membrane components with positive spontaneous curvature near the domain boundary^31^. This results in a sharp decrease of the boundary elastic energy allowing us to conclude that any membrane component with a strong positive spontaneous curvature should be line-active. This conclusion is quantitatively demonstrated for ganglioside GM1^31^.

It is conceivable that every molecule, which is able to make the transition from L_o_ to L_d_ phase more gradual, will partition to the domain boundary, thereby reducing the free energy of the interface. For example, if a transmembrane protein has a length, which is an average between L_o_ and L_d_ phase bilayer thicknesses, it should partition to the domain boundary. However, it is not immediately apparent whether other molecules, such as amphipathic peptides, will smooth the transition between the phases. In the present study, we analyze a potential affinity of different membrane components to the ordered domain boundary. The following components are considered: lipids possessing positive or negative spontaneous curvature; lipids composing monolayers with different equilibrium thickness; amphipathic and hydrophobic peptides; and transmembrane proteins of different molecular geometry. We consider membrane deformations induced by membrane inclusions and by the thickness mismatch at the domain boundary in the framework of the theory of elasticity of liquid crystals, adapted to lipid membranes^40^. This allowed us to qualitatively obtain the energy gain or penalty of placing the inclusion to the boundary as compared to its location in the bulk L_o_ or L_d_ phase. The dependence of the total elastic energy on the lateral position of the inclusion provides evidence of the line activity of different molecular components.

## Statement of the Problem

We consider a system of an ordered lipid domain, surrounding membrane and a membrane inclusion. The aim is to describe interactions, mediated by membrane elastic deformations, between the domain boundary and the inclusion.

### Elastic energy functional

We assume that the size of the lipid domain allows using a unidimensional approach, in which the domain boundary is considered as a straight line, i.e. the curvature of the boundary is neglected. Therefore, we calculate the interaction energy per unit length of the domain boundary. In order to obtain the total energy one needs to multiply the energy per unit length by the size of the inclusion in the direction along the boundary. For the unidimensional approach to be valid, the domain size should be much larger than the characteristic decay length of membrane deformations, *λ*. As *λ* ~ 1–2 nm^20,39,41^, the assumption of the straight boundary is valid for domains larger than about 10 nm in radius, i.e. almost for any observable domain^42^. In fact, a recent paper^41^, where the interaction between amphipathic peptides partially incorporated into a membrane is studied, suggests that the unidimensional approach could be applied even for domains less than 10 nm in radius; one needs only to know an effective size of the inclusion in order to obtain the total elastic energy^43^. Nevertheless, the examination of such small domains is beyond the scope of this paper. Thus, below we utilize the assumption that the system has a translational symmetry along the domain boundary. We introduce a Cartesian coordinate system *Oxyz*, the *y-*axis of which is directed along the domain boundary; *z*-axis is directed perpendicular to the membrane plane. We choose the position of the *Oyz*-plane in such a way as it intersects the membrane along the domain boundary of the lower monolayer. In this coordinate system, the system is translationally symmetric along the *y*-axis, and all deformations depend on the *x*-coordinate only, i.e. the system is effectively unidimensional.

Hereinafter, we follow the notations introduced by M. Hamm and M.M. Kozlov^40^. We characterize an average orientation of lipid molecules by a vector field of unit vectors **n** called directors^40,44^. The field of directors is defined on a surface lying inside the monolayer, referred to as a dividing surface. A field of unit vectors normal to the dividing surface is denoted by **N**. These normal vectors as well as directors are directed towards the monolayer interface of the membrane. We consider the following elastic deformations of the lipid monolayer: (1) splay, characterized by the splay modulus *B* and described by the divergence of the director along the dividing surface, div(**n**); (2) tilt, characterized by the tilt modulus *K*_*t*_ and described by a tilt vector **t** = **n − N**; (3) lateral compression/stretching, characterized by the modulus *K*_*a*_, and described by a relative expansion of the dividing surface area, *α* = (*a* − *a*_0_)/*a*_0_ (*a* is the current area per molecule, *a*_0_ is the initial area per molecule at the dividing surface). Besides, we take into account that the monolayer may be subjected to some lateral tension *σ*_0_. The energy contribution of the Gaussian curvature is zero because of the translational symmetry of the system. The free energy was calculated up to the second order in deformations, which are assumed to be small. In a quadratic approximation, the elastic energy of the monolayer can be expressed as^40,45,46^:

1 $$W=\int dS\,(\frac{B}{2}{({\rm{div}}{\bf{n}}+{J}_{0})}^{2}+\frac{{K}_{t}}{2}{{\bf{t}}}^{2}+\frac{{K}_{a}}{2}{(\alpha -{\alpha }_{0})}^{2}+\frac{{\sigma }_{0}}{2}{({\bf{g}}{\bf{r}}{\bf{a}}{\bf{d}}H)}^{2})$$

where *J*_0_ is the spontaneous curvature of the lipid monolayer; *α*_0_ = *σ*_0_/*K*_*a*_ is the equilibrium lateral stretching of the monolayer due to an imposed lateral tension; *H* is the distance between the dividing surface and the plane *Oxy*, measured along the normal to the plane. In Eq. (1), the integration is performed over the monolayer dividing surface. Deformations and elastic moduli are related to the specific dividing surface, where the energy contributions from splay and lateral compression/stretching deformations are independent. This surface is referred to as a neutral surface, which is shown experimentally to be located at the distance of about 0.7 nm from the outer surface of the monolayer, in the region of the junction between polar head groups and alkyl tails of lipid molecules^47^.

Generally, in the original theory of elasticity of lipid membranes introduced by W. Helfrich^44^ the lipid bilayer is supposed to be an infinitely thin film lacking any internal structure. In this approach, a membrane patch is considered as a single entity with one neutral surface. The applicability of this model is very limited. However, the attractiveness of the simplicity and efficiency of the Helfrich elastic energy functional motivated a number of successive modifications and generalizations. The most important generalization is the application of the functional separately to each lipid monolayer, rather than to the membrane as a whole. In this case, the deformations are related to a certain surface passing within the monolayer, and the deformation energy of the membrane is represented by the sum of deformation energies of two monolayers^48^. This modification was not rigorously validated, i.e., in fact, it was hypothesized *ad hoc*. However, the generalization of the Helfrich model was so natural that although never explicitly formulated, it was often successfully applied^49–53^. In experiments with non-lamellar (monolayer) inverted lipid phases it is shown that a lipid monolayer has its own neutral surface, lying in the region of junction of lipid polar heads and hydrophobic tails^47,54^. These findings justify to some extent the relation of the Helfrich functional^44^ and subsequently developed elastic functionals of lipid membranes^40,55^ separately to each lipid monolayer.

The compressibility modulus of the membrane is very large, about 10^10^ J/m^3^ (ref. ^56^). This allows imposing a local volumetric incompressibility constraint on monolayer deformations. Within the required accuracy, this constraint can be written as^40^:

2 $${h}_{c}=h-\frac{{h}^{2}}{2}{\rm{div}}{\bf{n}}-h\alpha $$

where *h*_*c*_ is the local thickness of the hydrophobic part of the monolayer; *h* is the thickness of the hydrophobic part of an undeformed monolayer. Below, we refer to *h*_*c*_ and *h* as the monolayer thicknesses, for simplicity.

Further, we indicate values corresponding to the upper monolayer by the index “*u*”, and those of the lower monolayer by the index “*l*”. Besides, we indicate parameters corresponding to the L_o_ phase by the index “*r*”, and parameters of the L_d_ surrounding membrane by the index “*s*”. Membrane shape is characterized by three functions: (1) the distance from the neutral surface of the upper monolayer to the plane *Oxy*, *H*_*u*_(*x*); (2) the distance from the neutral surface of the lower monolayer to the plane *Oxy*, *H*_*l*_(*x*); (3) the distance from the monolayer interface to the plane *Oxy*, *M*(*x*); all distances are measured along the normal to the *Oxy* plane. Substituting the local thickness of the monolayers into the incompressibility conditions (2), we obtain:

3 $$\begin{array}{rcl}{H}_{u}-M & = & {h}_{u}-\frac{{h}_{u}^{2}}{2}{\rm{div}}{{\bf{n}}}_{u}-{h}_{u}{\alpha }_{u},\\ M-{H}_{l} & = & {h}_{l}-\frac{{h}_{l}^{2}}{2}{\rm{div}}{{\bf{n}}}_{l}-{h}_{l}{\alpha }_{l}.\end{array}$$

Deformations are subjected to boundary conditions. Firstly, deformations should decay far from the domain boundary and from the inclusion, leading to the conditions:

4 $$\begin{array}{c}{\bf{n}}(\,\pm \,\infty )={\bf{0}},\,div({\bf{n}}(\,\pm \,\infty ))=0,\,{\bf{t}}(\,\pm \,\infty )={\bf{0}},\,a(\,\pm \,\infty )={a}_{0},\\ M(\,\pm \,\infty )=const,\,{H}_{u}(\,\pm \,\infty )=const,\,{H}_{l}(\,\pm \,\infty )=const.\end{array}$$

Secondly, director projections onto the coordinate axes and neutral surfaces are required to be continuous everywhere, except non-lipid undeformable membrane inclusions (as directors and neutral surfaces do not exist inside non-lipid inclusions). Membrane inclusions are formally characterized by specific boundary conditions, which depend on the type of inclusions and are described below.

We divide the membrane into three regions corresponding to the bilayer L_o_ phase, bilayer L_d_ phase and the transitional region between the L_o_ and L_d_ phases, where one monolayer is in the L_o_ state, and the other monolayer is in the L_d_ state. A membrane inclusion is taken into account by introduction of an additional, 4th region, where this inclusion is located; the width of the 4th region coincides with the width of the inclusion. We obtain the elastic energy of each region of the membrane minimizing the energy functional (1) with the condition of local volumetric incompressibility (2). The variation of the energy functional with respect to functions *n*_*u*_(*x*), *n*_*l*_(*x*), *H*_*u*_(*x*), *H*_*l*_(*x*), *M*(*x*) yields five simultaneous Euler-Lagrange differential equations. Because all regions of the membrane are conjugated only by the boundary conditions, Euler-Lagrange equations for these regions can be considered independently. The boundary conditions (4) and conditions of continuity of neutral surfaces and director projections are imposed on the solutions of the Euler-Lagrange equations. The details of the elastic energy calculation are presented in the Supplementary Information.

The shape of the monolayer interface, *M*(*x*), is common for energy functionals of upper and lower monolayers (see Eq. (3)). Thus, the opposing monolayers are not fully independent but strongly coupled via the interface *M*(*x*). For instance, deformations of the upper monolayer lead to the change of force and torque factors, such as div(**n**_**u**_), *α*_*u*_, and position of the neutral surface *H*_*u*_(*x*); this change immediately transmits to the monolayer interface as follows from the first equation of the volumetric incompressibility conditions (3); then, the alteration of *M*(*x*) results in the change of force and torque factors of the opposing (lower) monolayer according to the second equation of the volumetric incompressibility conditions (3). Effectively, conditions (3) ideally equilibrate at the interface *M*(*x*) forces and torques acting at neutral surfaces *H*_*u*_(*x*), *H*_*l*_(*x*) of two opposing monolayers. This balance results in a high symmetry of deformations arising in the monolayers (compare expressions for *H*_*u*_(*x*) and *H*_*l*_(*x*), as well as for *n*_*u*_(*x*) and *n*_*l*_(*x*) in Supplementary Eq. (S13)).

Note that the volumetric incompressibility conditions strictly constrain the deformations. The strictness implies that the forces ensuring the hold of the conditions are virtually infinite. Thus, the corresponding coupling of the opposing monolayers is not associated with any coupling energy. The experimentally determined coupling energy of two L_o_ monolayer domains into the bilayer L_o_/L_o_ domain^16^ effectively arises from the energy penalty of assembly of a “hybrid” L_o_/L_d_ bilayer. Recently, the origin of the penalty has been attributed to the unfavorable amplitude of thermal undulations of the “hybrid” L_o_/L_d_ bilayer as compared to two symmetric coexisting L_o_/L_o_ and L_d_/L_d_ membrane regions^18,19,21^. This coupling mechanism has entropic nature and cannot be described in terms of our elastic model, as the model does not explicitly consider thermal fluctuations.

### Boundary conditions for membrane inclusions

In this work, we consider the following membrane inclusions: amphipathic peptides, hydrophobic peptides, lipidic inclusions of non-zero spontaneous curvature and transmembrane proteins of different geometry. Below, we describe the boundary conditions for all types of inclusions.

#### Amphipathic peptides

**Figure 1:**
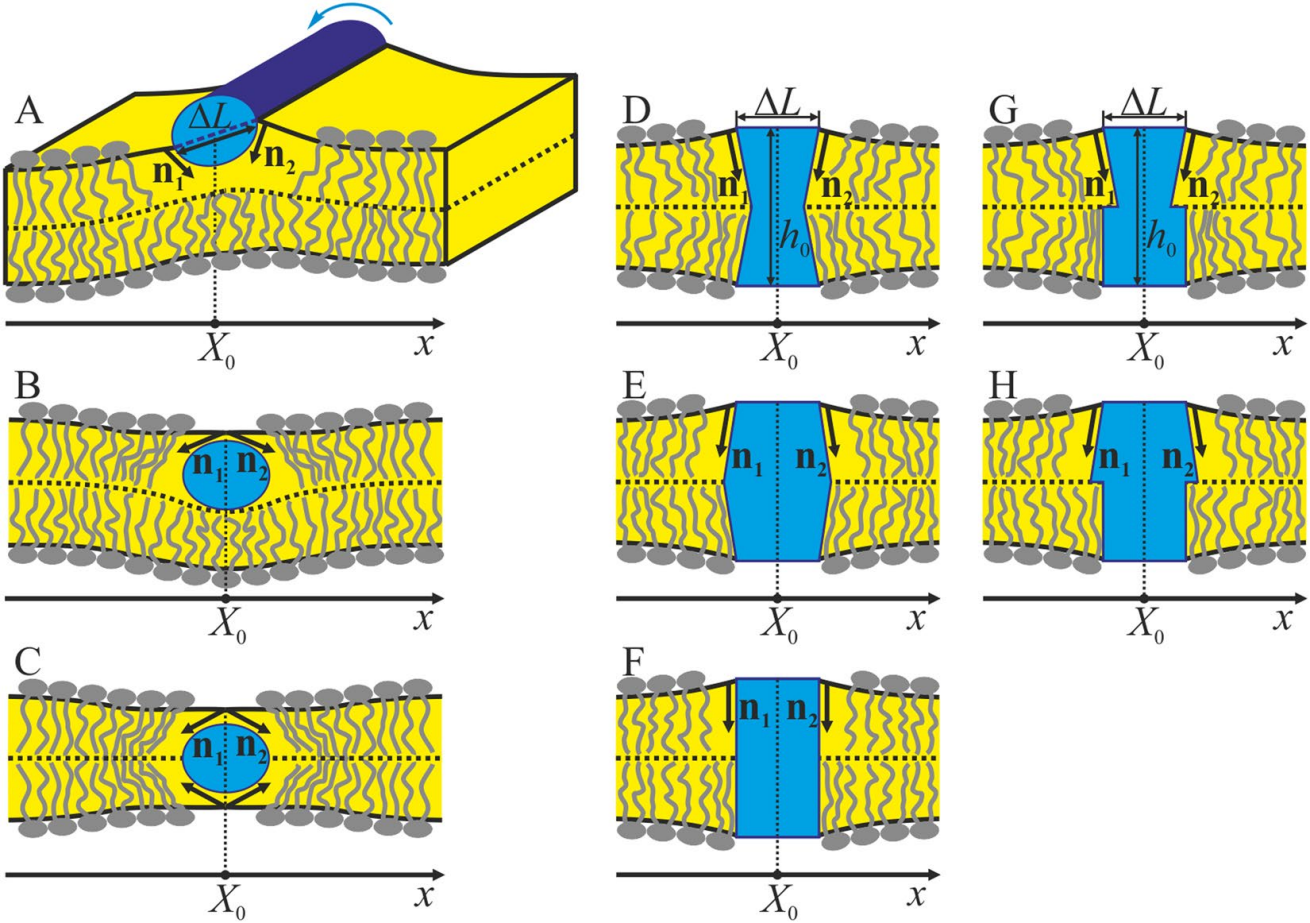
Boundary conditions induced by undeformable inclusions of width Δ*L*, the center of which is located at *x* = *X*_0_. (**A**) A shallowly inserted amphipathic peptide. The peptide pushes lipid heads apart, thereby inducing a non-zero jump in the boundary directors Δ**n** = **n**_2_ − **n**_1_ in the upper monolayer. The rotation of the peptide as a whole (designated by the blue arrow) around its longitudinal axis results in a relative shift of the upper monolayer neutral surface at the left and right boundaries of the peptide. (**B**) A hydrophobic peptide deeply incorporated into the upper monolayer. The peptide pushes lipid tails apart, inducing a non-zero jump in the boundary directors Δ**n** = **n**_2_ − **n**_1_ in the upper monolayer. The projection of Δ**n** onto the *x*-axis is positive, which is opposite to the case illustrated in panel A. The upper monolayer neutral surface remains continuous at the point *x* = *X*_0_. (**C**) A hydrophobic peptide deeply incorporated into the bilayer. The peptide pushes lipid tails apart, thereby inducing a jump in the boundary directors in both upper and lower monolayers, leaving the neutral surfaces continuous. (**D**–**F**) Symmetric transmembrane inclusions of the length *h*_0_ (*h*_0_ exceeds the bilayer thickness in the depicted cases). Inclusions have a fixed thickness and orientation of directors at their boundaries. (**D**) A hourglass-like inclusion. (**E**) A barrel-like inclusion. (**F**) A cylindrical inclusion. (**G**,**H**) Asymmetric transmembrane inclusions of the length *h*_0_ (*h*_0_ exceeds the bilayer thickness in the case depicted). (**G**) A semi-hourglass-like inclusion. (**H**) A semi-barrel-like inclusion. These inclusions are a combination of a cylindrical bottom part and a truncated cone top part.

A shallowly inserted amphipathic peptide moves apart polar lipid heads, inducing the tilt of adjacent lipids (Fig. 1A). In this case, there is a finite difference between directors at the left and right boundaries of the peptide, Δ**n** = **n**_2_ − **n**_1_.

When the peptide is located in the bulk phase (either L_o_ or L_d_), the value of a director jump can be qualitatively estimated from geometrical consideration in the following way^41,57^:

5 $$|\Delta {\bf{n}}|=\frac{\Delta L}{\sqrt{{(\Delta L/2)}^{2}+{(h/2)}^{2}}}$$

where Δ*L* is the width (diameter) of the peptide, *h* is the monolayer thickness of the corresponding phase (*h*_*r*_ or *h*_*s*_). When the peptide is located at the L_o_/L_d_ phase boundary, the director jump can be estimated as:

6 $$|\Delta {\bf{n}}|=\frac{\delta \Delta L}{\sqrt{{(\Delta L/2)}^{2}+{({h}_{r}/2)}^{2}}}+\frac{(1-\delta )\Delta L}{\sqrt{{(\Delta L/2)}^{2}+{({h}_{s}/2)}^{2}}}$$

where *δ* is the fraction of the peptide width (diameter), embedded into the L_o_ phase monolayer. The rotation of the peptide as a whole around its longitudinal axis (Fig. 1A) results in a relative shift of the upper monolayer neutral surface at the peptide left and right boundaries, thereby imposing the following boundary condition:

7 $${H}_{u}({X}_{0}+(DL/2))-{H}_{u}({X}_{0}-(DL/2))=DL|{{\bf{n}}}_{1}+{{\bf{n}}}_{2}|/2,$$

where *X*_0_ is the coordinate of the peptide center (peptide longitudinal axis); |**n**_1_ + **n**_2_|/2 is the average director characterizing the angle of the peptide rotation. We do not explicitly impose any constraints on the monolayer region opposing the adsorbed peptide, except the conditions of continuity of its directors and neutral surface at the interface with the adjacent bilayer. We therefore assume that the interactions between the peptide and hydrophobic lipid tails of the opposing monolayer do not create significant geometrical constraints.

The boundary conditions of Eqs. (5–7) are based on the simple geometrical consideration. The account for specific chemical and entropy-induced interactions between amphipathic peptides and lipids might be needed to quantitatively describe peptide-induced membrane shapes for particular peptides of well-defined chemical structure. A. J. Sodt and R.W. Pastor^58^ explicitly demonstrate the inadequacy of geometry-based boundary conditions for quantitative description of peptide-induced membrane curvature. By molecular dynamics, they show that the membrane curvature induced by the particular amphipathic peptide is about twice as high as predicted by the continuum model^59^. However, in our approach we do not consider particular peptides and their explicit chemical structure. In this concern, the boundary conditions of Eqs. (5, 6) should be treated as an approximate, qualitative estimation of the boundary director jump. For this reason, we varied the numerical value of $|\Delta {\bf{n}}|$ as a parameter in order to qualitatively describe the free energy landscape of the system.

#### Hydrophobic peptides

Deeply inserted hydrophobic peptide pushes hydrophobic lipid tails apart, leaving polar heads intact, i.e. the neutral surfaces remain continuous (*H*_*u*_(*X*_0_ − 0) = *H*_*u*_(*X*_0_ + 0), *H*_*l*_(*X*_0_ − 0) = *H*_*l*_(*X*_0_ + 0)). This induces the tilt of adjacent lipids, leading to a finite difference between directors at the same point *X*_0_ of the upper monolayer neutral surface: Δ**n** = **n**(*X*_0_ + 0) − **n**(*X*_0_ − 0) (Fig. 1B). When the peptide is incorporated symmetrically into the region of the intermonolayer surface (Fig. 1C), the jump in the boundary directors Δ**n** occurs in both upper and lower monolayers, whereas both neutral surfaces remain continuous. The difference Δ**n** depends on the peptide width Δ*L*, on the depth of its incorporation and on the monolayer thickness *h* in a somewhat unobvious manner. Thus, we use the value of |Δ**n**| as a numeric parameter to obtain the elastic energy of the membrane, induced by the peptide. As in the case of amphipathic peptides, when we consider the hydrophobic peptide in the upper monolayer (the case depicted in Fig. 1B) we do not explicitly impose additional constraints on the opposing monolayer.

#### Transmembrane proteins

Transmembrane proteins are assumed to have a fixed thickness and a fixed orientation of directors at their boundaries (Fig. 1D–H).

#### Lipidic inclusions

To describe lipidic inclusions, we introduce an additional membrane region, consisting of a monolayer stripe of width Δ*L* with prescribed elastic parameters. This allows taking into account lipidic inclusions with various spontaneous curvatures *J*_0_ and equilibrium monolayer thickness *h*. At the boundary of the stripe, we impose conditions of continuity of directors and neutral surfaces.

### Elastic parameters

In order to quantitatively illustrate the obtained results, we use the following values of membrane elastic parameters: splay moduli of the liquid-ordered and liquid-disordered monolayer *B*_*r*_ = 20 *k*_*B*_*T* and *B*_*s*_ = 10 *k*_*B*_*T* (*k*_*B*_*T* ≈ 4 × 10^−21^ J), respectively^60–62^; monolayer thicknesses of the liquid-ordered and liquid-disordered monolayer *h*_*r*_ = 1.8 nm and *h*_*s*_ = 1.3 nm, respectively^32,34^; the lateral tension (per monolayer) *σ*_0_ = 0.025 *k*_*B*_*T*/nm^2^ ≈ 0.1 mN/m. For simplicity of the illustration, spontaneous curvatures of the liquid-ordered and liquid-disordered monolayers are assumed to be zero, *J*_*r*_ = *J*_*s*_ = 0. Tilt moduli are the same in both phases, *K*_*t*_^*r*^ = *K*_*t*_^*s*^ = *K*_*t*_ = 40 mN/m; the tilt modulus is approximately equal to the surface tension at the oil/water interface, and therefore it weakly depends on the chemical nature and physical state of lipids^40^. Typical values of lateral compression/stretching moduli are relatively large, especially for membranes containing an appreciable amount of cholesterol^61^. Thus, the energy stored in this deformation is relatively small and weakly sensitive to the exact value of the lateral compression/stretching modulus. For this reason, we use equal values of moduli for monolayers of both phases, *K*_*a*_^*r*^ = *K*_*a*_^*s*^ = *K*_*a*_ = 120 mN/m (ref. ^60^). The width of all membrane inclusions, both deformable (lipid monolayer) and undeformable (peptides and transmembrane proteins) is assumed to be equal to Δ*L* = 1.3 nm, i.e. approximately to the diameter of a single *α*-helix.

We assume the monolayers in the transition region between L_o_ and L_d_ phases to have the same properties as the corresponding monolayers in the bulk phases. One may argue that this region should be considered as a quasi-third bilayer phase with different elastic parameters. However, molecular dynamics simulations of asymmetric “hybrid” bilayers, composed of one L_o_ and one L_d_ monolayers, show that initial properties of constituent monolayers changes but slightly^63^. In particular, in such “hybrid” bilayer the area per lipid molecule in L_o_ monolayer increased by about 5%, as compared to the symmetric L_o_ bilayer. On the contrary, the area per lipid molecule in L_d_ monolayer decreased by about 7% from this value in the symmetric L_d_ bilayer. Nevertheless, in the asymmetric bilayer the area per lipid molecule in L_d_ and L_o_ phases still differs by ∼31%, although in the symmetric bilayer the difference of the area per molecule in L_d_ and L_o_ phases is ∼44% (ref. ^63^). In the work ref. ^64^ the authors consider highly asymmetric vesicles made from POPC and di-palmitoyl-phosphatidyl-choline (DPPC). The inner monolayer is enriched in POPC and is in a liquid-disordered state. The outer monolayer contains an appreciable amount of DPPC and is mainly in a gel phase. Wherein, the area per POPC molecule in the inner monolayer remains the same as in the symmetric L_d_ POPC bilayer, while in the outer monolayer the area per DPPC molecule increases by about 10% as compared to the gel phase DPPC bilayer^64^. Thus, we consider the change of elastic properties of L_o_ and L_d_ monolayers within the “hybrid” bilayer as relatively small, and attribute to the monolayers in the transition region between bilayer L_o_ and L_d_ phases the same properties as for corresponding monolayers in the bulk phases.

## Results

### Equilibrium structure of the domain boundary

**Figure 2:**
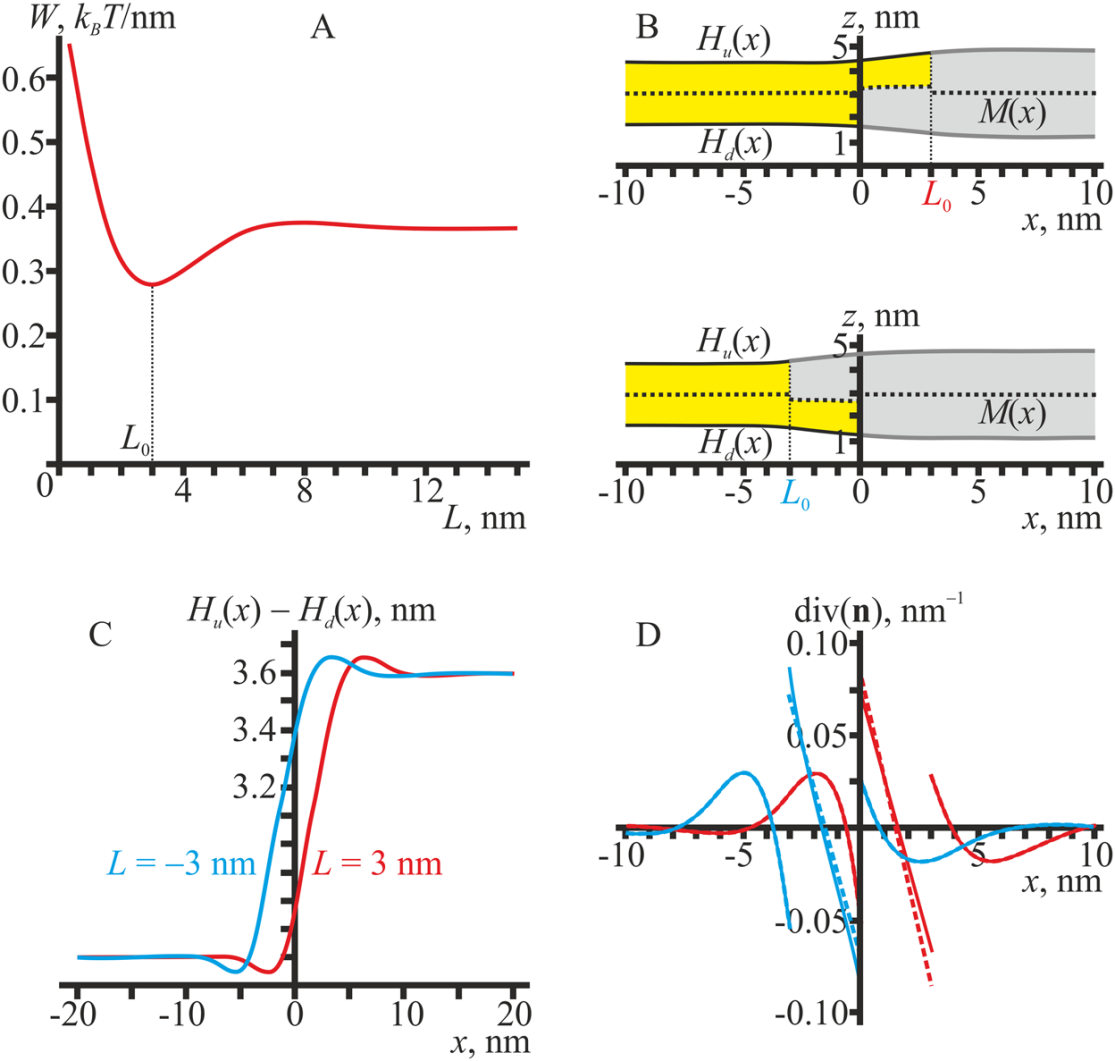
The equilibrium structure of the domain/surrounding membrane boundary. (**A**) The dependence of the elastic energy on the coordinate of the L_o_/L_d_ phase boundary in the upper monolayer. The energy is minimal when the L_o_/L_d_ phase boundaries in the upper and lower monolayers are relatively shifted by *L*_0_ = 3 nm. (**B**) Membrane shapes corresponding to the optimal relative shift of the boundaries of monolayer domains in the upper and lower monolayers. Top — *L* = 3 nm; bottom — *L* = −3 nm. The neutral surfaces of the L_d_ monolayers (yellow) are drawn as a solid black line, the neutral surfaces of the L_o_ monolayers (grey) — as a solid thick grey line, the monolayer interface *M*(*x*) — as a dotted black line. (**C**) The local thickness of the bilayer, *H*_*u*_(*x*) − *H*_*l*_(*x*), corresponding to *L* = 3 nm (red curve) and *L* = −3 nm (blue curve). The thickness varies from 2*h*_*s*_ = 2.6 nm at *x* → −∞ to 2 *h*_*r*_ = 3.6 nm at *x* → +∞. (**D**) The dependence of the splay (div(**n**)) on the *x* coordinate. Red curves — *L* = 3 nm; blue curves — *L* = −3 nm. The splay in the upper monolayer is shown by solid lines; the splay in the lower monolayer — by dashed lines.

Firstly, we consider the equilibrium structure of the domain boundary in the absence of inclusions. For the chosen set of parameter values (see above), the elastic energy of the boundary (per unit length) is minimal and equal to ∼0.27 *k*_*B*_*T*/nm ≈ 1.1 pN, when the relative shift of the boundaries of the L_o_ monolayers is equal to *L*_0_ = 3 nm (Fig. 2A). The value of 1.1 pN agrees well with the experimentally determined value of the line tension of the L_o_ domain boundary^17^. The dependence of the elastic energy *W* on the position of the L_o_/L_d_ phase boundary in the upper monolayer is symmetric, i.e. *W*(*L*) = *W*(−*L*).

Shapes of the membrane in two optimal configurations corresponding to *L* = ± *L*_0_ = ± 3 nm are presented in Fig. 2B (top — *L* = 3 nm, bottom — *L* = −3 nm). There is a transitional region of width *L*_0_ = 3 nm between L_o_ and L_d_ phases. In this region, the bilayer is “hybrid”: one monolayer is in the L_o_ state, and the other one is in the L_d_ state. In our elastic model, the domain boundary structure is determined mainly by the thickness mismatch between the bilayers of L_o_ and L_d_ phases. This thickness mismatch is considered as a driving force for the lateral sorting of transmembrane proteins having different lengths of their hydrophobic transmembrane domains^65–67^. The intermediate region provides the gradual change of the bilayer thickness from 2 *h*_*r*_ in the L_o_ phase to 2*h*_*s*_ in the L_d_ phase (Fig. 2C), thereby potentially participating in such sorting by allowing the protein to occupy the lateral position where the thickness of the bilayer is optimal.

In equilibrium, both L_o_ and L_d_ bilayers are flat far from the boundary, i.e. they have zero curvature. This is formally described by the condition div(**n**(±∞)) = 0. In the vicinity of the boundary, deformations arising in order to compensate for the thickness mismatch lead to a non-zero splay, i.e. div(**n**) ≠ 0. The dependence of the local splay div(**n**) of each monolayer on the *x*-coordinate is presented in Fig. 2D. The local splay varies drastically in the intermediate region and its vicinity. This potentially provides a driving force for the lateral sorting of membrane inclusions preferring a particular curvature, such as amphipathic and hydrophobic peptides or non-bilayer lipids (i.e. forming monolayer with non-zero spontaneous curvature).

### Lipidic inclusion of zero spontaneous curvature

**Figure 3:**
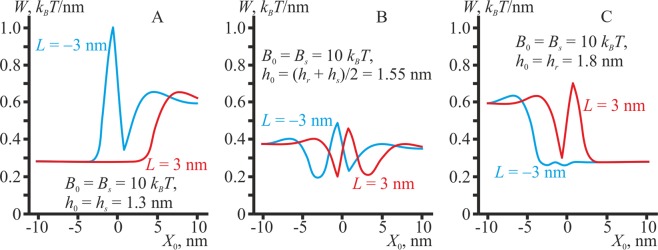
The dependence of the membrane elastic energy on lateral position *X*_0_ of the center of the lipidic inclusion of width Δ*L* = 1.3 nm, possessing a zero spontaneous curvature. (**A**) Monolayer thickness *h*_0_ = *h*_*s*_ = 1.3 nm. (**B**) Monolayer thickness *h*_0_ = (*h*_*r*_ + *h*_*s*_)/2 = 1.55 nm. (**C**) Monolayer thickness *h*_0_ = *h*_*r*_ = 1.8 nm. Red curves correspond to *L* = 3 nm; blue curves — to *L* = −3 nm. Curves are obtained for the lipidic inclusion splay modulus *B*_0_ = *B*_*s*_ = 10 *k*_*B*_*T*; for *B*_0_ = *B*_*r*_ = 20 *k*_*B*_*T* the curves are almost the same.

We consider the dependence of the elastic energy on the position of a lipidic inclusion with a zero spontaneous curvature (Fig. 3).

**Figure 4:**
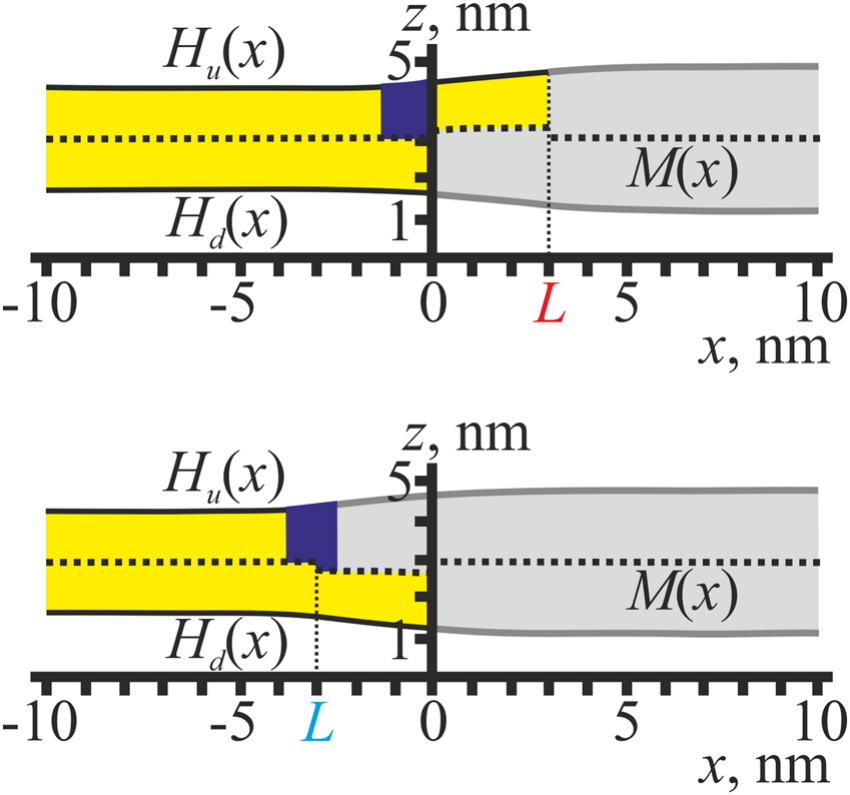
The equilibrium shapes of the membrane for optimal positions of lipidic inclusions of intermediate thickness, *h*_0_ = 1.55 nm. Top — *L* = 3 nm; bottom — *L* = −3 nm. Optimal positions correspond to the global minima of the elastic energy (Fig. 3B). The L_d_ monolayers are shown in yellow, and their neutral surfaces are drawn as solid black lines; the L_o_ monolayers are shown in grey, and their neutral surfaces are drawn as solid thick grey lines; the monolayer interface *M*(*x*) is drawn as a dotted black line. Lipidic inclusions are shown in dark blue.

Independently from the sign of *L* = ±3 nm, both thin (*h*_0_ = *h*_*s*_ = 1.3 nm) and thick (*h*_0_ = *h*_*r*_ = 1.8 nm) lipidic inclusions prefer to distribute to the bulk of the L_d_ and L_o_ phases, respectively, as upon such distribution the monolayer thickness perfectly fits the thickness of the inclusion, thus allowing the thickness mismatch to be minimized. Stripe of the lipid monolayer of the intermediate thickness *h*_0_ = (*h*_*r*_ + *h*_*s*_)/2 prefers to be located inside the intermediate region or close to it, providing two sharp local minima of similar depth of the elastic energy. The minima correspond to the location of the lipidic inclusion close to the symmetric bilayer phase, either the L_d_ (global minima) or the L_o_ (local minima); the energy minima depth is about 0.17 *k*_*B*_*T*/nm. The curves calculated for the splay modulus of the lipid monolayer inclusion *B*_0_ = *B*_*r*_ = 20 *k*_*B*_*T* and *B*_0_ = *B*_*s*_ = 10 *k*_*B*_*T* are virtually the same. Membrane shapes for the optimal position of the lipid monolayer of thickness *h*_0_ = 1.55 nm are shown in Fig. 4 in the cases of *L*_0_ = 3 nm (top) and *L*_0_ = −3 nm (bottom) for *B*_0_ = *B*_*s*_ = 10 *k*_*B*_*T* only; in the case of *B*_0_ = *B*_*r*_ = 20 *k*_*B*_*T* the shapes are very similar.

### Lipidic inclusion of non-zero spontaneous curvature

In the case of non-zero spontaneous curvature of the lipidic inclusion, the pattern of its interaction with the domain boundary becomes more complex, as two factors work simultaneously: the optimization of the thickness mismatch and minimization of the curvature stress. We consider the dependence of the membrane elastic energy on the lateral position *X*_0_ of the center of the stripe of the lipid monolayer of width Δ*L* = 1.3 nm, possessing either positive (*J*_0_ = +0.25 nm^−1^) or negative (*J*_0_ = −0.25 nm^−1^) spontaneous curvature, in the case of different equilibrium thicknesses *h*_0_. The splay modulus of the lipidic inclusion is assumed to be *B*_0_ = 10 *k*_*B*_*T*; parameters of the energy profiles appeared to be weakly dependent on the exact value of the modulus, and profiles calculated for the case of *B*_0_ = 20 *k*_*B*_*T* almost overlap with those obtained for *B*_0_ = 10 *k*_*B*_*T*.

**Figure 5:**
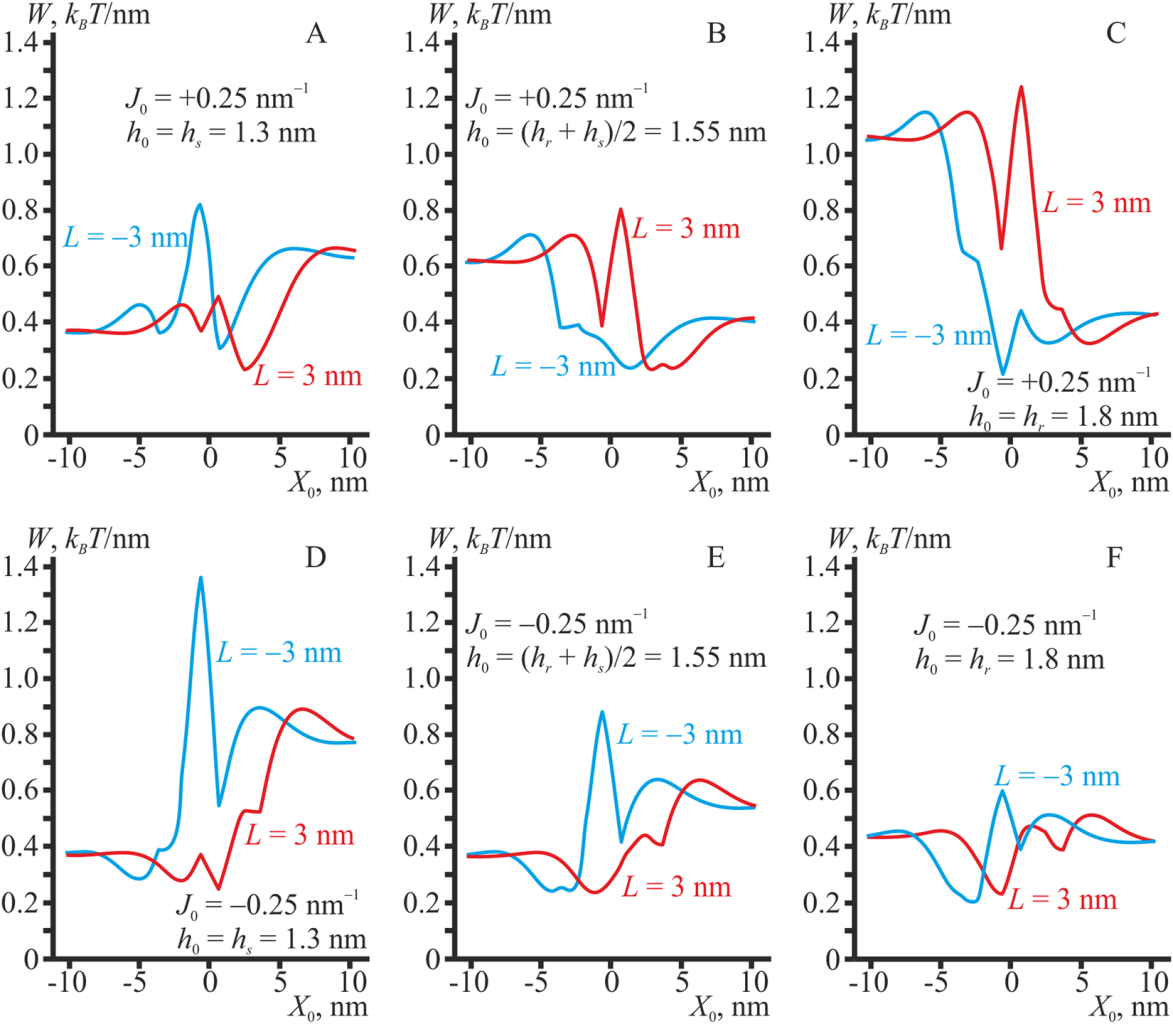
The dependence of the membrane elastic energy on the lateral position *X*_0_ of the lipidic inclusion of width Δ*L* = 1.3 nm, possessing a positive (top raw, *J*_0_ = +0.25 nm^−1^) or negative (bottom raw, *J*_0_ = –0.25 nm^−1^) spontaneous curvature. The thickness of the lipid monolayer: (**A,D**) *h*_0_ = *h*_*s*_ = 1.3 nm; (**B,E**) *h*_0_ = (*h*_*r*_ + *h*_*s*_)/2 = 1.55 nm; (**C,F**) *h*_0_ = *h*_*r*_ = 1.8 nm. Red curves correspond to *L* = 3 nm; blue curves — to *L* = −3 nm. The curves are obtained for the lipid monolayer splay modulus *B*_0_ = *B*_*s*_ = 10 *k*_*B*_*T*; for *B*_0_ = *B*_*r*_ = 20 *k*_*B*_*T* the curves virtually coincide.

The most pronounced preference for the L_o_/L_d_ phase boundary has a thick lipidic inclusion (*h*_0_ = 1.8 nm) of a negative spontaneous curvature (*J*_0_ = −0.25 nm^−1^): the depth of the energy minima equals about Δ*W* ≈ 0.2–0.25 *k*_*B*_*T*/nm for *L*_0_ =  ±3 nm (Fig. 5F). In the case of the positive spontaneous curvature (*J*_0_ = +0.25 nm^−1^), the preference of the thick monolayer for the boundary is manifested only for *L*_0_ = −3 nm (Fig. 5C): the depth of the energy minimum is Δ*W* ≈ 0.2 *k*_*B*_*T*/nm. Lipid monolayer of the intermediate thickness *h*_0_ = (*h*_*r*_ + *h*_*s*_)/2 = 1.55 nm always has a preference for the intermediate region, as this optimally relaxes elastic stresses arising from both the thickness mismatch and curvature stress; depths of the corresponding energy minima are approximately Δ*W* ≈ 0.15 *k*_*B*_*T*/nm. The value of the spontaneous curvature influences only the exact optimal position of the lipidic inclusion inside the intermediate region (compare locations of the energy minima in Fig. 5B,E). In cases of the thick (*h*_0_ = 1.8 nm) lipid monolayer with the positive spontaneous curvature (*J*_0_ = +0.25 nm^−1^) and *L*_0_ = +3 nm or thin (*h*_0_ = 1.3 nm) lipid monolayer with any spontaneous curvature (*J*_0_ = ±0.25 nm^−1^) and *L*_0_ = −3 nm, there is only a weak affinity to the L_o_/L_d_ phase boundary (Fig. 5C,D). In these cases, location of the lipid monolayer stripe inside the intermediate region is favorable, as this allows its spontaneous curvature and local geometric curvature to be optimally adjusted. However, the thickness of the surrounding monolayer in the intermediate region is non-optimal, as it differs from the thickness of the lipid monolayer. Thus, the thickness mismatch neutralizes the energy gain from the adjustment of the spontaneous and local geometric curvatures, thus leading to only weak preference of the lipidic inclusion for the domain boundary.

### Shallow peptide inclusion

**Figure 6:**
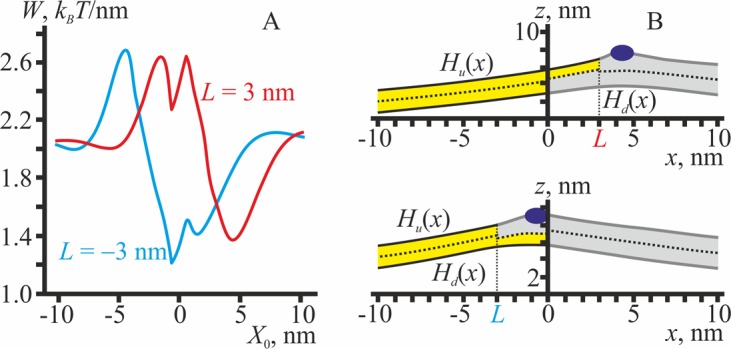
(**A**) The energy of membrane deformations induced by the shallowly inserted amphipathic peptide. (**B**) Membrane shapes in two optimal configurations of the domain boundary with the incorporated peptide. The peptide is shown as a dark blue ellipse.

As an example of the shallowly inserted peptide inclusion, we consider an *α*-helical amphipathic peptide, the upper side surface of which is hydrophilic, and faces water, while the opposite side surface is hydrophobic and is partially immersed into the upper lipid monolayer. The axis of the *α*-helix is assumed to be directed along the domain boundary, i.e. parallel to the *y*-axis (Fig. 1A). The shallowly inserted *α*-helical amphipathic peptide has a strong preference for the domain boundary. The energy gain of the peptide relocation from the bulk of either the L_o_ or L_d_ phase to the vicinity of the boundary is about 0.6–0.7 *k*_*B*_*T*/nm (Fig. 6A).

In optimal configurations (corresponding to the global minima of the elastic energy), the peptide is located in the L_o_ monolayer either in the intermediate region (Fig. 6B, bottom) or in the bilayer L_o_ phase close to the intermediate region (Fig. 6B, top). The optimal position of the peptide roughly corresponds to the regions where div(**n**_**u**_) is negative (Fig. 2D), as it fits best the director jump at the peptide boundaries (Fig. 1A).

### Deep peptide inclusion

**Figure 7:**
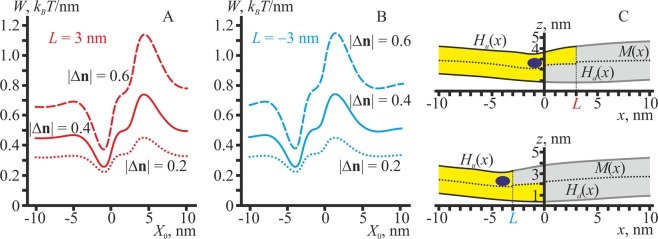
The energy of membrane deformations induced by the hydrophobic peptide deeply inserted into the upper monolayer. (**A**) *L* = +3 nm; (**B**) *L* = −3 nm. Dotted curves — the director jump at the peptide boundaries |Δ**n**| = |**n**_2_ − **n**_1_| = 0.2 (see Fig. 1B); solid curves — |Δ**n**| = 0.4; dashed curves — |Δ**n**| = 0.6. (**C**) Membrane shapes in two optimal configurations of the domain boundary with the hydrophobic peptide incorporated into the upper monolayer. The boundary director jump |Δ**n**| = 0.4 that corresponds to the solid curves in panels A, B. Top — *L* = +3 nm; bottom — *L* = −3 nm. The peptide is shown as a dark blue ellipse.

A hydrophobic peptide can be deeply incorporated into the lipid monolayer. This causes the inclination of hydrophobic lipid tails in the vicinity of the peptide, while lipid heads remain almost intact (Fig. 1B), resulting in the director jump at the peptide boundaries. The deeply incorporated peptide has a strong preference for the L_o_/L_d_ phase boundary for any value of the jump in the director in the upper monolayer (Fig. 7A,B), although the energy gain upon the distribution to the optimal location strongly depends on the director jump value.

In optimal configurations (corresponding to the global minima of the elastic energy, Fig. 7A,B), the peptide is located in the bilayer L_d_ phase, close to the intermediate region (Fig. 7C). In the optimal position, the peptide generally tends to distribute to the regions where div(**n**_**u**_) is highly positive (Fig. 2D), as it fits best the director jump at the peptide boundaries (Fig. 1B).

### Peptide inclusion at the intermonolayer surface

**Figure 8:**
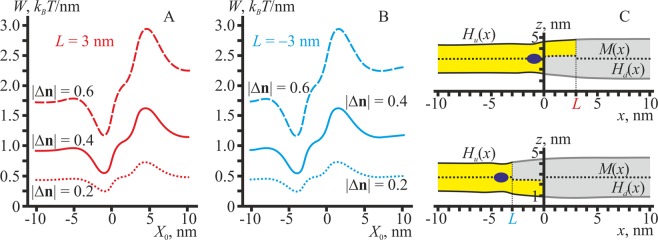
The energy of membrane deformations induced by the hydrophobic peptide incorporated into the region of the monolayer interface. (**A**) *L* = +3 nm; (**B**) *L* = −3 nm. Dotted curves — the director jump at the peptide boundaries |Δ**n**| = |**n**_2_ − **n**_1_| = 0.2 (see Fig. 1B); solid curves — |Δ**n**| = 0.4; dashed curves — |Δ**n**| = 0.6. (**C**) Membrane shapes in the two optimal configurations of the domain boundary with the hydrophobic peptide incorporated into the region of the monolayer interface. The boundary director jump |Δ**n**| = 0.4 that corresponds to the solid curves in panels A, B. Top — *L* = +3 nm; bottom — *L* = −3 nm. The peptide is shown as a dark blue ellipse.

A hydrophobic peptide can be incorporated into the region of the monolayer interface. In this case, hydrophobic lipid tails are distorted in both upper and lower monolayers, while lipid head regions remain intact (Fig. 1C). Such peptides prefer the domain boundary for any value of the director jump at the peptide boundaries (Fig. 8A,B), although the energy gain upon the distribution to the optimal location depends on the jump value.

In the optimal configurations, corresponding to the global minima of the elastic energy (Fig. 8A,B), the peptide is located in the L_d_ bilayer phase, close to the intermediate region (Fig. 8C). In the optimal position, the peptide generally tends to distribute to the regions where div(**n**_**u**_) is highly positive in both upper and lower monolayers (Fig. 2D), as it fits best the director jump at the left and right boundaries of the peptide (Fig. 1C). Because locations of the regions where the div(**n**_**u**_) is highly positive almost coincide in the opposing monolayers, incorporation of the symmetric peptide into the monolayer interface results in about two times deeper energy minima as compared to the case of incorporation of the hydrophobic peptide into the upper monolayer only (compare Figs. 7A,B and 8A,B), for the same values of the boundary directors.

### Symmetric transmembrane inclusion

**Figure 9:**
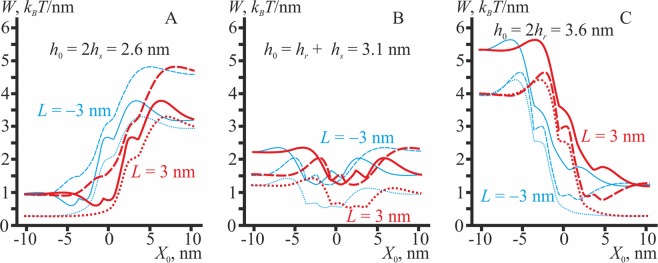
The energy of membrane deformations induced by transmembrane symmetric inclusions of different length *h*_0_. (**A**) *h*_0_ = 2*h*_*s*_; (**B**) *h*_0_ = *h*_*r*_ + *h*_*s*_; (**C**) *h*_0_ = 2 *h*_*r*_. Dotted curves correspond to cylindrical inclusions (Δ**n**_*x*_ = 0); dashed curves — to hourglass-like inclusions (Δ**n**_*x*_ = −0.4); solid curves — to barrel-like inclusions (Δ**n**_*x*_ = +0.4). Red curves — *L* = +3 nm; blue curves — *L* = −3 nm.

As a symmetric transmembrane inclusion we consider a cylindrical protein with the vertical boundary directors satisfying Δ**n**_*x*_ = (**n**_2_ − **n**_1_)_*x*_ = 0 (the subscript “*x*” means projection of the vector onto the *x*-axis) (Fig. 1F), a hourglass-like protein, Δ**n**_*x*_ = (**n**_2_ − **n**_1_)_*x*_ = −0.4 (Fig. 1D), and a barrel-like protein, Δ**n**_*x*_ = (**n**_2_ − **n**_1_)_*x*_ = +0.4 (Fig. 1E). We also consider different lengths of the transmembrane part (*h*_0_ = 2*h*_*s*_ (Fig. 9A); *h*_0_ = *h*_*s*_ + *h*_*r*_ (Fig. 9B); *h*_0_ = 2 *h*_*r*_ (Fig. 9C)).

All types of symmetric transmembrane inclusions preferentially distribute to the intermediate region, if their length fits the thickness of bilayer in this region, i.e. *h*_0_ = *h*_*r*_ + *h*_*s*_ (Fig. 9B). In this case, the lateral redistribution is driven mainly by the thickness mismatch optimization, which is naturally achieved in the intermediate region. The cylindrical inclusion preferentially distributes to the intermediate region only when *h*_0_ = *h*_*r*_ + *h*_*s*_ (Fig. 9B, dotted curve). In contrast, if *h*_0_ = 2 *h*_*r*_ or *h*_0_ = 2*h*_*s*_, the cylindrical inclusion prefers the bulk phases, where the thickness mismatch is minimal (L_o_ or L_d_, respectively), demonstrating no affinity to the domain boundary (Fig. 9A, C dotted curves). Long hourglass-like inclusion (*h*_0_ = 2 *h*_*r*_) subjects the membrane to the minimal elastic stress when it is located in the bilayer L_o_ phase adjacent to the intermediate region (dashed curves in Fig. 9C), while short hourglass-like inclusions (*h*_0_ = 2*h*_*s*_) show no affinity to the boundary, preferring to distribute into the bulk of the L_d_ phase (dashed curves in Fig. 9A). On the contrary, long barrel-like inclusions (*h*_0_ = 2 *h*_*r*_) do not prefer the domain boundary (solid curves in Fig. 9C), while short inclusions (*h*_0_ = 2*h*_*s*_) have a relatively high affinity to the bilayer L_d_ region adjacent to the intermediate region (solid curves in Fig. 9A). Generally, the lateral distribution of all symmetric transmembrane inclusions is driven mainly by the thickness mismatch optimization. Besides, non-cylindrical inclusions (hourglass or barrel-like) tend to distribute to the membrane regions where div(**n**_**u**_) and div(**n**_**d**_) fit best the jump in the directors at the left and right boundaries of the inclusion, i.e. where div(**n**_**u,d**_) <0 for hourglass-like inclusions and div(**n**_**u,d**_) >0 for barrel-like inclusions. As inclusions have the finite width Δ*L* = 1.3 nm, they physically cannot occupy the point where the maximum (barrel-like inclusions) or minimum (hourglass-like inclusion) of div(**n**_**u,d**_) is achieved, thus distributing to the neighborhood of this point (Fig. 2D).

### Asymmetric transmembrane inclusion

**Figure 10:**
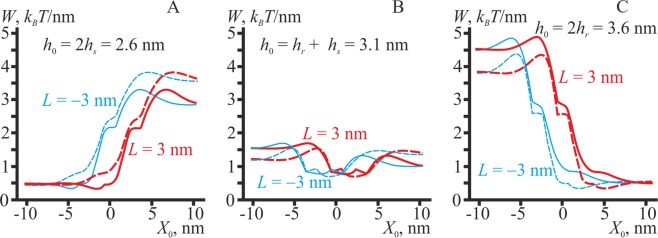
The energy of membrane deformations induced by transmembrane asymmetric inclusions of different length *h*_0_. (**A**) *h*_0_ = 2*h*_*s*_; (**B**) *h*_0_ = *h*_*r*_ + *h*_*s*_; (**C**) *h*_0_ = 2 *h*_*r*_. Dashed curves correspond to semi-hourglass-like inclusions (Δ**n**_*x*_^*u*^ = –0.4, Δ**n**_*x*_^*l*^ = 0); solid curves — to semi-barrel-like inclusions (Δ**n**_*x*_^*u*^ = +0.4, Δ**n**_*x*_^*l*^ = 0). Red curves — *L* = +3 nm; blue curves — *L* = −3 nm.

We consider semi-hourglass-like proteins as asymmetric transmembrane inclusions, the boundary directors of which satisfy the following relations: Δ**n**_*x*_^*u*^ = (**n**_2_ − **n**_1_)_*x*_ = −0.4, Δ**n**_*x*_^*l*^ = 0 (the superscript “*u*” means the director of the upper monolayer, “*l*” — of the lower monolayer) (Fig. 1G), and semi-barrel-like proteins, Δ**n**_*x*_^*u*^ = (**n**_2_ − **n**_1_)_*x*_ = +0.4, Δ**n**_*x*_^*l*^ = 0 (Fig. 1H) of different length of the transmembrane part (*h*_0_ = 2*h*_*s*_ (Fig. 10A); *h*_0_ = *h*_*s*_ + *h*_*r*_ (Fig. 10B); *h*_0_ = 2 *h*_*r*_ (Fig. 10C)).

All types of asymmetric transmembrane inclusions preferentially distribute to the intermediate region, if their length fits the thickness of bilayer in this region, i.e. *h*_0_ = *h*_*r*_ + *h*_*s*_ (Fig. 10B). In this case, the lateral redistribution is driven mainly by the thickness mismatch optimization, which is naturally achieved in the intermediate region. Besides, the intermediate region is weakly preferred by short semi-barrel-like (solid curves in Fig. 10A) and long semi-hourglass-like inclusions (dashed curves in Fig. 10C). On the contrary, short semi-hourglass-like and long semi-barrel-like inclusions preferentially distribute to the bulk of the L_d_ or L_o_ phase, respectively (Fig. 10A, dashed curves and Fig. 10C, solid curves). The lateral distribution of asymmetric transmembrane inclusions is qualitatively similar to the lateral distribution of corresponding symmetric transmembrane inclusions (compare Fig. 9 and Fig. 10).

## Discussion

In the present work, we considered interactions, mediated by membrane elastic deformations, between membrane inclusions and the boundary of liquid-ordered domain. Deformations arising at the domain boundary make the local curvature of monolayer neutral surfaces non-zero, allowing the membrane inclusion to choose the optimal position, in which the curvature stress is minimal. In addition, different thicknesses of the L_o_ and L_d_ bilayers influence the lateral distribution of transmembrane proteins and lipidic inclusions of various lengths. In the vicinity of the domain boundary, the equilibrium thickness of the membrane gradually changes from 2*h*_*s*_ in the L_d_ phase to 2 *h*_*r*_ in the L_o_ phase (Fig. 2C). The local curvature is equal to zero in the bulk of both phases and changes sharply and non-monotonously at the boundary (Fig. 2D). Besides, we considered elastic moduli of peptides and proteins as infinitely large, i.e. these inclusions were treated as undeformable. This provides an additional driving force for the preferential lateral distribution of undeformable inclusions to the domain boundary: inclusions may substitute for deformed membrane regions, thus effectively nullifying the elastic energy stored in these regions, as no elastic energy may be stored in undeformable inclusions. This driving force is counteracted by specific boundary conditions on membrane deformations imposed by inclusions. Generally, the dependence of the elastic energy on the position of membrane inclusions can be very complex, especially for membrane inclusions possessing both a spontaneous curvature and a thickness mismatch (Figs. 9, 10).

Our calculations are based on the assumption of translational symmetry of the system along the domain boundary, i.e. along the *y*-axis. For this reason, we present the elastic energy per unit length of the boundary (*k*_*B*_*T*/nm) rather than the absolute energy (*k*_*B*_*T*). In order to obtain absolute energy values, one needs to multiply the energy per unit length by the length of the membrane inclusion along the domain boundary. Recently, we have demonstrated that for exponentially decaying membrane elastic deformations the energy of interaction between membrane inclusions of various sizes and mutual orientations can be well approximated by the unidimensional potential multiplied by an effective length, which slightly exceeds the actual size of the inclusion in the plane of the membrane by a factor of ∼1.3–2 (ref. ^41^). It means that in order to obtain the absolute energy gain upon the optimal localization of, e.g., an amphipathic peptide of an actual length of ∼5 nm (in the plane of the membrane) along the domain boundary one should multiply the calculated energy per unit length of the boundary, ∼0.6 *k*_*B*_*T*/nm (Fig. 6A), by the effective length: ∼(1.3–2) × 5 nm ≈8 nm, which yields ∼5 *k*_*B*_*T*. Similarly, in order to obtain the energy gain upon the optimal localization of lipidic inclusions (e.g., a monolayer stripe of a non-zero spontaneous curvature) at the domain boundary one should multiply the energy per unit length of the boundary by the domain perimeter.

According to our calculations, all membrane inclusions having a non-zero spontaneous curvature, but lacking any preference for a bilayer thickness, should predominantly distribute to the L_o_/L_d_ phase boundary because only in this region the local curvature differs from zero. For this reason, the domain boundary is strongly preferred by amphipathic peptides (Fig. 6) as well as by hydrophobic peptides incorporated both into the monolayer (Fig. 7) and into the region of the monolayer interface (Fig. 8). Some indirect experimental evidences of this attraction towards the domain boundary are present in the literature. For example, such an attraction should give rise to a clusterization of amphipathic molecules at the domain boundary. The clusterization is indicated in ref. ^68^, where the enhancement of pore formation by the amphipathic peptide melittin in membranes with coexisting L_o_ and L_d_ phases is shown. As the pore formation by amphipathic peptide is a cooperative process, involving several peptide molecules, the enhancement of the poration in the presence of L_o_/L_d_ phase boundary may point to the local increase of the melittin surface concentration in the vicinity of the boundary. In addition, in ref. ^69^ experiments show the tendency of the protein Equinatoxin II to concentrate at the domain boundary. Another example is the decrease of the line tension at the L_o_ domain boundary: in ref. ^70^ authors demonstrate that the amphipathic peptide Bax-α5 destroys a circular shape of ordered domains. In addition, it was experimentally shown that the fusion peptide of the HIV gp41 protein provides the membrane fusion most effectively when L_o_ and L_d_ phases co-exist in the target membrane^25,26^. As the effective fusion requires orchestrated action of several proteins, its enhancement for phase-separated membranes may indicate the local enrichment of the fusion peptide at the L_o_/L_d_ phase boundary. Shallowly incorporated amphipathic peptides or amphipathic parts of proteins participate in numerous cell processes, in particular, in membrane fusion, fission and poration^71–75^. We hypothesize that the L_o_/L_d_ phase boundary can drive a local enrichment of hydrophobic and amphipathic peptides, facilitating their organization to a highly efficient cooperative unit.

A local enrichment of the L_o_/L_d_ phase boundary by lipids possessing a positive spontaneous curvature was recently indicated as a mechanism of a line activity of ganglioside GM1^31^. GM1 introduced into the membrane in small amounts (~1 mol %) induces a decreases in the average size of the L_o_ domains, which is consistent with the drastic drop of the domain boundary energy^27,31,76^. Our elastic model describes both effects: GM1 local enrichment and drop of the boundary energy (Fig. 5A–C). Typically, a local increase in the concentration of lipids having a positive spontaneous curvature leads to the formation of through pores in membranes^45^, even in the presence of cholesterol^51,77^, which has a highly negative spontaneous curvature^78,79^. However, the enrichment of ganglioside GM1 at the domain boundary is not accompanied by the pore formation^27,31^, while the enrichment of lysolipids produced by phospholipase A2 indeed results in the membrane poration^77^. This difference in the pore-forming activity may arise from different local concentrations of GM1 and lysolipids in these experiments. Besides, GM1 has a large polar part in comparison with lysophosphatidylcholines. Large polar heads may hinder GM1 molecules reorientation, which is an essential stage of the hydrophilic pore formation^45,46^.

The distribution of GM1 molecules in a two-phase gel/liquid system is studied in ref. ^80^, where authors demonstrate that GM1 preferentially incorporates into the gel phase. Although the energy functional (1) is valid only for laterally fluid monolayers, i.e. for vanishing lateral shear mode of deformations, qualitatively the gel phase can be described as a fluid membrane possessing much larger elastic moduli in comparison with the “common” fluid phase. From results depicted in Fig. 5, we can conclude that the monolayer thickness of a lipidic inclusion with a spontaneous curvature is the main factor determining the inclusion localization in the L_o_ or L_d_ phase. Qualitatively, the same can be concluded for the gel/liquid systems. Actually, the authors in ref. ^80^ explain GM1 lateral distribution via a hydrocarbon chain length matching between GM1 and gel phase lipids, and the same explanation holds within the framework of our elastic model. However, in the course of the phase transition, GM1 molecules may be trapped in the gel phase, and hence they can be unable to occupy the optimal location at the domain boundary. The distribution of GM1 in the coexisting L_o_/L_d_ phases is studied in ref. ^81^, where, with the help of cholera toxin labeling, it is shown that GM1 is preferentially located in the L_o_ phase. Again, chain length matching between L_o_ phase lipids and GM1 can explain this fact. Although our theory predicts that a global minimum of the energy of the system is achieved when GM1 is located at the L_o_ domain boundary, it does not mean that all GM1 molecules should necessarily gather at this boundary region, as the depth of the corresponding energy minimum is not large. From Fig. 5C, it follows that the energy difference between the GM1 localization in L_o_ and L_d_ phases is about 0.75 *k*_*B*_*T*/nm, while the difference between the energy values in the global minimum and in the case of GM1 located in L_o_ phase is about 0.15–0.2 *k*_*B*_*T*/nm. Because the second value (∼0.2 *k*_*B*_*T*/nm) is three times smaller than the first one (0.75 *k*_*B*_*T*/nm), the difference in GM1 concentrations at the domain boundary and in the bulk of the L_o_ phase might be beyond the resolution of the fluorescence methods used in ref. ^81^, although the difference of GM1 concentrations in the bulk of L_o_ and L_d_ phases may be still observable. In our recent work on the GM1 line activity, we obtained that the L_o_/L_d_ boundary line tension depends non-monotonously on the GM1 concentration at high cholesterol content in the membrane. In order to explain this effect, we had to assume that the boundary has a finite capacity for GM1, and the excess of GM1 distributes to the bulk L_o_ phase^31^. Such mode of GM1 lateral distribution may explain low contrast between GM1 concentrations at the domain boundary and in the bulk L_o_ phase, as observed by fluorescent microscopy^81^.

As for transmembrane inclusions, we showed that their distribution between the L_o_ and L_d_ phases is modulated by two factors: their length and orientation of the boundary directors. The evidence that the length of the transmembrane protein can determine its preference for a certain lipid phase follows from experiments described in ref. ^82^, where the decrease in the length of the transmembrane protein LAT (the linker for activation of T cells) by each amino acid leads to a reduced by ~5% association of this protein with the liquid-ordered phase. There are plenty of classical papers on the distribution of transmembrane proteins between gel and fluid phases^83–86^. In these experiments, the proteins visible by electron microscopy are excluded from the gel phase. It is conceivable that the mismatch between the thickness of the gel bilayer and the length of transmembrane proteins is highly unfavorable because of large elastic moduli of the gel phase in comparison with the fluid phase.

A lot of experiments demonstrate partitioning of either peripheral^87–95^ or transmembrane^65,82,96–109^ proteins between lipid phases. A comprehensive review of these works is presented in ref. ^110^. As underlined in this review, there are no general insights that can be applied to predict the partitioning properties of a given protein. In fact, some post-translational modifications of proteins, such as palmitoylation and myristoylation, may be anticipated as determinants of the preferential partitioning of proteins into the L_o_ phase due to a high affinity of the attached saturated lipids to the more ordered environment. However, the situation is somewhat trickier as there are proteins with both palmitoylation and myristoylation, which nevertheless manifest preference for the L_d_ phase^88,91^. In addition, alterations in a conformation of acylated proteins can influence their partitioning^111^. On the other hand, proteins anchored by glycophosphatidylinositol (GPI), or so-called GPI-anchored proteins, always partition to the L_o_ phase due to the chemical affinity of GPI to this phase^110^. A thorough review of how lipid modifications influence the partitioning of proteins between phases is given in ref. ^112^. Among transmembrane proteins, LAT appears to be the most studied protein with regard to the partitioning between the phases. It is known that the single-pass transmembrane domain (tLAT) of LAT accounts for its partitioning characteristics^82^. As already mentioned, the reduction in length of tLAT diminishes its raft association^82^. In addition, molecular dynamics simulations of tLAT in membranes show the preference of both depalmitoylated and palmitoylated tLAT to the L_d_ phase^113^, which is consistent with experiments on GUVs^114^. Simulations also predict the affinity of tLAT to the L_o_/L_d_ interfaces^113,115,116^. Interestingly, energy profiles obtained with the help of potential of mean force calculations^113^ are similar to those for hour-glass transmembrane inclusions (Fig. 9A), predicted by our model. Experimentally, such an attraction to the domain boundary is indicated for the HIV receptor CCR5^26^.

We emphasize that the presence of a local minimum on the dependence of the elastic energy on the lateral position of an inclusion does not necessarily mean that the inclusion will always stay at that particular location. If an ensemble of inclusions has reached its thermal equilibrium, the Boltzmann distribution should be satisfied. Consider, for example, short (*h*_0_ = 2.6 nm) barrel-like (Δ**n**_*x*_ = +0.4) inclusions, the energy profile of which is illustrated by the solid red curve in Fig. 9A, of an effective lateral length (measured along the domain boundary) of 1 nm. The fulfillment of the Boltzmann distribution implies that these inclusions should be depleted in the bulk L_o_ phase by a factor of about exp{∼2 *k*_*B*_*T*/nm·1 nm/(*k*_*B*_*T*)} ≈ exp(2) ≈ 7.4 as compared to the concentration in the L_d_ phase (here 2 *k*_*B*_*T*/nm is the difference of the system elastic energy when the inclusion is located in the L_o_ and L_d_ phases, respectively, and 1 nm is the effective lateral size of the inclusion). Besides, the L_o_/L_d_ phase boundary should be enriched by the inclusions by a factor of about exp(0.4 *k*_*B*_*T*/nm·1 nm/(*k*_*B*_*T*)) ≈ exp(0.4) ≈ 1.5 (here 0.4 *k*_*B*_*T*/nm is the depth of the energy local minimum of the red solid curve at *X*_0_ ≈ −2 nm, Fig. 9A), as compared to the surface concentration of the inclusions in the L_d_ phase. In the work ref. ^113^ on molecular modeling of multiple tLAT inserted into the membrane manifesting the L_o_/L_d_ phase coexistence it is demonstrated that tLAT first distributes to the L_o_/L_d_ phase boundary, and then after saturation of the boundary, predominantly distributes to the bulk of the L_d_ phase.

In contrast to experiments with giant unilamellar vesicles (GUV), palmitoylated tLAT is distributed approximately equally between the phases in giant plasma membrane vesicles (GPMVs)^82,107^. As an explanation for this difference between the experiments on GPMVs and GUVs, a smaller difference of lipid packing in the L_o_ and L_d_ phases in GPMVs as compared to GUVs is suggested in ref. ^113^ and supported by the corresponding experiments^117,118^. This explanation can be further elaborated within the framework of our model. A recent study^119^ indicates that membrane bending moduli correlate with the lipid packing parameter. Thus, it is likely that the difference between the bending moduli of phases is higher in GUVs than in GPMVs. Therefore, as predicted by our model, the presence of any source of membrane deformations in the L_o_ phase of GUVs is more unfavorable than in GPMVs and may explain the strong exclusion of tLAT from the L_o_ phase in GUVs. We note that palmitoylation also influences tLAT’s preference to the phases: depalmitoylation of tLAT results in its exclusion from the L_o_ phase in GPMVs^107^. However, palmitoylation of tLAT may create kinks on the cytoplasmic side of tLAT^113^, which, within the framework of our model, corresponds to the transformation of tLAT towards the semi-barrel-like configuration, and, hence, to its enhanced affinity to the L_o_ phase (Fig. 10B). It should also be noted that protein-lipid interactions may play a crucial role in the partitioning of proteins as shown experimentally by alteration of the protein accessible surface area (ASA)^120^. Therefore, the interplay between lipid-lipid and protein-lipid interactions as well as membrane deformations may determine the partitioning of tLAT.

Three key determinants of the L_o_/L_d_ phase partitioning of transmembrane proteins are indicated in ref. ^120^: (i) post-translational modification (i.e. palmitoylation and myristoylation) favors distribution to the L_o_ phase; (ii) accessible surface area of the protein, which correlates to some extent with the transmembrane domain (TMD) diameter (a smaller ASA favors the distribution to the L_o_ phase); (iii) the length of the TMD (a smaller length favors the distribution to the L_d_ phase). These determinants seem to be independent in the sense that their variation results in approximately additive effects on the protein partitioning. For example, a decrease of the TMD length with simultaneous decrease of ASA results in almost unaltered partitioning of the modified LAT as compared to the wild type^120^. A variation of the determinants should lead to alteration of the protein-induced deformations of the membrane. Thus, in the framework of our elastic approach, the protein-induced deformations may be considered as a single quantitative effective combination of three determinants. Although this combination is not sufficient to definitely judge on the protein partitioning, as the elastic approach ignores direct chemical protein-lipid and lipid-lipid interactions, nevertheless, it may be useful for predicting the partitioning in the cases when the elastic driving forces are obviously large. For example, a characteristic energy of a pairwise repulsion of the saturated palmitoyl acyl chain from the unsaturated oleoyl acyl chain may be estimated as about 1 *k*_*B*_*T* (ref. ^121^). Thus, if the depth of the elastic energy minimum at a certain position of a palmitoylated protein is larger than 1 *k*_*B*_*T*, the protein lateral distribution should be mainly determined by induced membrane deformations rather than by its preference to the L_o_ phase due to unfavorable interactions of palmitoyl with unsaturated lipids enriched in the L_d_ phase.

The targeting to the L_o_ phase of both peripheral and integral membrane proteins is sometimes considered to be determined by specific amino acid sequences known as CRAC — cholesterol-recognizing amino acid consensus. The general structure of the CRAC motif is [−L/V−(X)(1−5)−Y−(X)(1−5)−R/K−], where (X)(1–5) is 1 to 5 arbitrary amino acids^122,123^. The CRAC motif is assumed to bind cholesterol. As the L_o_ phase is relatively enriched in cholesterol, such binding can drive CRAC-containing proteins to the L_o_ phase^123^. However, there is experimental evidence that the L_o_ phase is rather moderately enriched in cholesterol^14^: in model lipid membranes formed from DOPC, DPPC and cholesterol the relative enrichment of cholesterol in the L_o_ phase as compared to the L_d_ phase is only ∼1.5:1 at 20–25 °C. In membranes composed of cholesterol, DSPC and either DOPC or POPC the maximal partition coefficient of cholesterol between L_o_ and L_d_ phases is about 3 (∼8% of cholesterol in L_d_ phase vs. ∼25% — in L_o_ phase)^124^. Such an enrichment is observed for a relatively low total content of cholesterol in membranes (<25%); for higher concentrations of cholesterol, the partition coefficient decreases. This means that the cholesterol binding cannot lead to a highly specific targeting of CRAC-containing proteins to the L_o_ phase, because cholesterol distribution between the coexisting phases is not so contrast. Moreover, experiments with plasma membranes show a nearly uniform lateral distribution of cholesterol, and the sphingomyelin-rich domains are not enriched by cholesterol as compared to the surrounding membrane^1,2^. Besides, even if the amplified concentration of cholesterol exists in the L_o_ phase, it will not necessarily result in an increased concentration of the CRAC-cholesterol complex in the L_o_ phase, because the preference of this complex for the L_o_ phase might be weaker than that of free cholesterol. Actually, the binding constant of CRAC-proteins for cholesterol in the L_o_ phase might be higher than in the L_d_ phase. However, there is no relevant information in the literature. For these reasons, the mechanisms of the targeting of CRAC-containing proteins to the L_o_ phase and their influence on raft-dependent cell processes remain obscure. Based on the results obtained, we can propose several hypotheses about the CRAC motif action mechanisms. First, the motif is amphipathic, as it simultaneously includes charged (R/K), aromatic (Y) and hydrophobic (L/V) amino acids. Hence, the presence of this motif in a peptide increases its amphipathicity. Our model predicts a strong preference of peripheral amphipathic peptides to the L_o_/L_d_ phase boundary (Fig. 6A). If cholesterol actually binds to the CRAC motif, a peptide will transform into a predominantly hydrophobic peptide of the shallow incorporation (Fig. 1B). However, this transformation should not influence the distribution of the peptide, as a shallowly incorporated hydrophobic peptide also has a strong preference for the L_o_/L_d_ phase boundary (Fig. 7). As for integral proteins, the CRAC motif should be located in the region of the junction between polar heads and hydrophobic tails of lipids, i.e. close to the monolayer neutral surface, because this motif includes both charged and hydrophobic amino acids. The binding of cholesterol will likely increase the volume of the corresponding hydrophobic part of the protein-cholesterol complex, thereby slightly modulating its distribution. For example, cylindrical proteins will transform into semi-barrel-like ones (Fig. 1H). In the case of proteins having a long transmembrane domain (dotted curves in Fig. 9C), this transformation may lead to a slight preference for the L_o_/L_d_ interface (dashed curves in Fig. 10C).

Our elastic model predicts that for major types of membrane inclusions the L_o_/L_d_ phase boundary serves as a universal attractor inducing the local enrichment of inclusions. The enrichment, in turn, leads to the alteration of the boundary energy, thereby inducing the size redistribution of domains and variation of the total length of domain boundaries, resulting in the alteration of local concentrations of membrane inclusions. Thus, we predict feedback in the system of L_o_ domains and laterally arranged membrane inclusions. We hypothesize that such behavior may be used by living cells to finely regulate the size distribution of membrane domains by small amounts of line-active membrane components.

## Supplementary information

Supplementary information.
