# Supplementary material for: Elastic deformations mediate interaction of the raft boundary with membrane inclusions leading to their effective lateral sorting
Source: Sci Rep. 2020 Mar 5;10:4087. doi: 10.1038/s41598-020-61110-2 (PMC7058020; doi:10.1038/s41598-020-61110-2)
Supplement: Supplementary file 1 — Supplementary information. [file 41598_2020_61110_MOESM1_ESM.doc]

**Supporting Material**

**Elastic deformations mediate interaction of the raft boundary with membrane inclusions leading to their effective lateral sorting**

Konstantin V. Pinigin, Oleg V. Kondrashov, Irene Jiménez-Munguía, Veronika V. Alexandrova, Oleg V. Batishchev, Timur R. Galimzyanov, Sergey A. Akimov

We consider a system of an ordered lipid domain, surrounding membrane and a membrane inclusion. The aim is to describe interactions, mediated by membrane elastic deformations, between the domain boundary and the inclusion.

For the elastic energy calculations, the membrane is divided into several regions: the bilayer L_d_ phase, the L_o_/L_d_ transition region, the bilayer L_o_ phase, and the region where the membrane inclusion is located. The bilayers in L_o_ and L_d_ phases are symmetric in the sense that their constituent monolayers have the same elastic parameters, whereas the bilayer in the L_o_/L_d_ transition region is asymmetric. In addition, the inclusions such as amphipathic peptides or lipid monolayers with non-zero spontaneous curvature require special consideration of the opposing monolayer. The total energy of the system is the sum of the contributions from all of the regions. Because the regions are conjugated only by the boundary conditions, the Euler-Lagrange equations for these regions can be considered independently. Below, we provide the general solutions of these equations corresponding to various cases.

**Elastic energy of asymmetric bilayer**

The membrane shape is characterized by three functions: 1) distance from the neutral surface of the upper monolayer to the plane *Oxy*, *H_u_*(*x*); 2) distance from the neutral surface of the lower monolayer to the plane *Oxy*, *H_l_*(*x*); 3) distance from the monolayer interface to the plane *Oxy*, *M*(*x*); all distances are measured along the normal to the *Oxy* plane. In these notations, a local thickness of the upper and lower monolayer is *h_u_* = *H_u_* – *M* and *h_l_* = *M* – *H_l_*, respectively. Substituting the local thickness of the monolayers into the incompressibility conditions (2), we obtain:

$\begin{matrix} H_{u}-M=h_{u}-\frac{h_{u}^{2}}{2}\text{div} \mathbf{n}_{u}-h_{u}\alpha_{u}, \\ M-H_{l}=h_{l}-\frac{h_{l}^{2}}{2}\text{div} \mathbf{n}_{l}-h_{l}\alpha_{l}. \end{matrix}$ (S1)

These conditions allow expressing the compression/stretching deformation field through the others:

$\begin{matrix} \alpha_{u}=1-\frac{h_{u}}{2}\mathrm{div}\mathbf{n}_{u}+\frac{1}{h_{u}}M-\frac{1}{h_{u}}H_{u}, \\ \alpha_{l}=1-\frac{h_{l}}{2}\mathrm{div}\mathbf{n}_{l}-\frac{1}{h_{l}}M+\frac{1}{h_{l}}H_{l}. \end{matrix}$ (S2)

Because of the translational symmetry of the system, all vector variables can be replaced to their projections onto the *x*-axis: **n** → *n_x_* = *n*; **N** → *N_x_* = *N*; the divergence and gradient become derivatives with respect to *x*: div(**n**) → *dn*/*dx*, **grad**(*H*) → *dH*/*dx*. Taking into account equations (S2) and relations *N_u_* = *dH_u_*(*x*)/*dx*, *N_l_* = –*dH_l_*(*x*)/*dx*, which are valid within the required accuracy, from equation (1) we obtain the following expression for the elastic energy functional:

$\begin{matrix} W=\int dS_{u}\left\{\frac{B_{u}}{2}\left(n'_{u}+J_{u}\right)^{2}+\frac{K_{t}^{u}}{2}\left(n_{u}-H'_{u}\right)^{2}+\frac{\sigma_{0}^{u}}{2}\left(H'_{u}\right)^{2}+\frac{K_{a}^{u}}{2h_{u}^{2}}\left(h_{u}-\frac{h_{u}^{2}}{2}n'_{u}+M-H_{u}-h_{u}\alpha_{0}\right)^{2}\right\} \\ +\int dS_{l}\left\{\frac{B_{l}}{2}\left(n'_{l}+J_{l}\right)^{2}+\frac{K_{t}^{l}}{2}\left(n_{l}+H'_{l}\right)^{2}+\frac{\sigma_{0}^{l}}{2}\left(H'_{l}\right)^{2}+\frac{K_{a}^{l}}{2h_{l}^{2}}\left(h_{l}-\frac{h_{l}^{2}}{2}n'_{l}-M+H_{l}-h_{l}\alpha_{0}\right)^{2}\right\}. \end{matrix}$ (S3)

In this expression, a prime superscript denotes the derivative with respect to *x*. The integrals in equations (S3) are performed over the neutral surfaces of the upper and lower monolayers, respectively. The translational symmetry allows considering the elastic energy per unit length along the domain boundary, and the neutral surface area elements can be expressed as *dS_u_* = *Y*⋅*dx* = *dx*, *dS_l_* = *Y*⋅*dx* = *dx*, where *Y* = 1 is a unit length along the domain boundary.

Hamm and Kozlov show that the tilt modulus is approximately equal to the surface tension at the water/oil interface, and therefore weakly dependent on the chemical nature of lipids^40^. The compression/stretching modulus is usually relatively high, *K_a_* >> *K_t_*, and hence the energy stored in this deformation is relatively small. Besides, in conventional cellular and model systems, the lateral tension is the same in both monolayers. Therefore, for the sake of simplicity we assume *K_t_^u^* = *K_t_^l^* = *K_t_*; *K_a_^u^* = *K_a_^l^* = *K_a_*; *σ*_0_*^u^* = *σ*_0_*^l^* = *σ*_0_. However, these conditions do not imply that the bilayer is fully symmetric, as the monolayers can have different thicknesses (*h_u_* ≠ *h_l_*), splay moduli (*B_u_* ≠ *B_l_*), and spontaneous curvatures (*J_u_* ≠ *J_l_*).

The variation of the energy functional (S3) with respect to the functions *n_u_*, *n_d_*, *H_u_*, *H_l_*, *M* results in five Euler-Lagrange equations:

$\left\{\begin{matrix} \left(l_{u}^{2}+\frac{h_{u}^{2}A}{4}\right)n''_{u}-n_{u}-\frac{A}{2}M'+\left(1+\frac{A}{2}\right)H'_{u}=0, \\ \left(l_{l}^{2}+\frac{h_{l}^{2}A}{4}\right)n''_{l}-n_{l}+\frac{A}{2}M'-\left(1+\frac{A}{2}\right)H'_{l}=0, \\ \left(1+\frac{A}{2}\right)n'_{u}-\left(1+\sigma\right)H''_{u}+\frac{A}{h_{u}^{2}}\left(H_{u}-M-h_{u}\left(1-\alpha_{0}\right)\right)=0, \\ \left(1+\frac{A}{2}\right)n'_{l}+\left(1+\sigma\right)H''_{l}-\frac{A}{h_{l}^{2}}\left(H_{l}-M+h_{l}\left(1-\alpha_{0}\right)\right)=0, \\ n'_{u}-n'_{l}+\frac{2}{h_{u}^{2}}\left(H_{u}-M-h_{u}\left(1-\alpha_{0}\right)\right)+\frac{2}{h_{l}^{2}}\left(H_{l}-M+h_{l}\left(1-\alpha_{0}\right)\right)=0, \end{matrix}\right.$ (S4)

where *l_u_* = (*B_u_*/*K_t_*)^1/2^, *l_l_* = (*B_l_*/*K_t_*)^1/2^, *A* = *K_a_*/*K_t_*, *σ* = σ_0_/*K_t_*. Note that the spontaneous curvatures *J_u_*, *J_l_* do not enter the Euler-Lagrange equations. By linear transformations, equations (S4) can be reduced to a single equation for *H_u_*(*x*):

$\begin{matrix} \left(4l_{u}^{2}l_{l}^{2}\left(h_{u}^{2}+h_{l}^{2}\right)+A\left(h_{u}^{4}l_{l}^{2}+h_{l}^{4}l_{u}^{2}\right)\right)H_{u}^{\left(8\right)}+ \\ +\frac{4A\left(l_{u}^{2}h_{l}^{2}+l_{l}^{2}h_{u}^{2}-2l_{u}^{2}l_{l}^{2}\right)-A\sigma\left(l_{u}^{2}+l_{l}^{2}\right)\left(h_{u}^{2}+h_{l}^{2}\right)-4\sigma\left(l_{u}^{2}+l_{l}^{2}\right)\left(h_{u}^{2}+h_{l}^{2}\right)}{1+\sigma}H_{u}^{\left(6\right)}+ \\ +\frac{4\sigma^{2}\left(h_{u}^{2}+h_{l}^{2}\right)+4A\left(l_{u}^{2}+l_{l}^{2}\right)+4A\sigma\left(2l_{u}^{2}+2l_{l}^{2}-h_{u}^{2}-h_{l}^{2}\right)}{\left(1+\sigma\right)^{2}}H_{u}^{\left(4\right)}-\frac{8A\sigma}{\left(1+\sigma\right)^{2}}H''_{u}+\alpha_{0}Z=0, \end{matrix}$ (S5)

where *Z* is the known bulky coefficient, which depends on elastic parameters of the system: *l_u_*, *l_l_*, *h_u_*, *h_l_*, *A*, *σ*. The general solution of equation (S5) can be written as follows:

$H_{u}\left(x\right)=\alpha_{0}Z\frac{\left(1+\sigma\right)^{2}}{16A\sigma}x^{2}+c_{1}e^{-q_{1}x}+c_{2}e^{q_{1}x}+c_{3}e^{-q_{2}x}+c_{4}e^{q_{2}x}+c_{5}e^{-q_{3}x}+c_{6}e^{q_{3}x}+c_{7}x+c_{8}$ (S6)

where *c*_5_, *c*_6_, *c*_7_, *c*_8_ are real constant coefficients, and *c*_1_, *c*_2_, *c*_3_, *c*_4_ are complex constant coefficients, which depend on the boundary conditions (4)–(7) appended by the requirement that all functions are real at any real *x*; *q*_3_ is real, and *q*_1_, *q*_2_ are conjugate complex roots of the bicubic characteristic polynomial of equation (S5). The function *H_d_*(*x*) has the form:

$\begin{matrix} H_{l}\left(x\right)=c_{1}k_{1}e^{-q_{1}x}+c_{2}k_{1}e^{q_{1}x}+c_{3}k_{2}e^{-q_{2}x}+c_{4}k_{2}e^{q_{2}x}+c_{5}k_{3}e^{-q_{3}x}+c_{6}k_{3}e^{q_{3}x}+ \\ +c_{7}x+c_{8}-h_{u}-h_{d}+\alpha_{0}Z\left(k_{4}+\frac{\left(1+\sigma\right)^{2}}{16A\sigma}x^{2}\right), \end{matrix}$ (S7)

where *k*_1_, *k*_2_, *k*_3_, *k*_4_ are known constant coefficients, which depend on elastic parameters of monolayers. The coefficients are very bulky, so we do not present them here. We denote five Euler-Lagrange equations (S4) as *E*_1_, *E*_2_, *E*_3_, *E*_4_, *E*_5_. The linear combination (*E*_5_(1 + *A*/2) + *E*_4_ – *E*_3_) results in the equation, which contains only *M*(*x*) and *H_u_*(*x*), *H_l_*(*x*), *H_u_*″(*x*), *H_l_*″(*x*) and does not contain directors. Solving this equation for *M*(*x*), we obtain:

$M\left(x\right)=\frac{h_{u}^{2}h_{l}^{2}\left(1+\sigma\right)}{2\left(h_{u}^{2}+h_{l}^{2}\right)}\left(H''_{u}+H''_{l}\right)+\frac{h_{l}^{2}H_{u}+h_{u}^{2}H_{l}}{h_{u}^{2}+h_{l}^{2}}-\frac{h_{u}h_{l}\left(h_{u}-h_{l}\right)\left(1-\alpha_{0}\right)}{h_{u}^{2}+h_{l}^{2}},$ (S8)

which allows obtaining the explicit expression for *M*(*x*) upon substitution of *H_u_*(*x*) and *H_l_*(*x*) from equations (S6) and (S7). Further, the linear combination $\left(\frac{4E_{1}}{4l_{u}^{2}+Ah_{u}^{2}}-\frac{2E'_{3}}{2+A}\right)$ results in the equation, which contains only *n_u_*(*x*), and *H_u_*′(*x*), *H_u_*′″(*x*), *M*′(*x*). Solving this equation for *n_u_*(*x*), we get:

$n_{u}\left(x\right)=\frac{\left(1+\sigma\right)\left(4l_{u}^{2}+Ah_{u}^{2}\right)}{2\left(2+A\right)}H'''_{u}+\frac{2\left(h_{u}^{2}+A\left(h_{u}^{2}-l_{u}^{2}\right)\right)}{h_{u}^{2}\left(2+A\right)}H'_{u}-\frac{A\left(h_{u}^{2}-2l_{u}^{2}\right)}{h_{u}^{2}\left(2+A\right)}M'.$ (S9)

The linear combination $\left(\frac{4E_{2}}{4l_{l}^{2}+Ah_{l}^{2}}-\frac{2E'_{4}}{2+A}\right)$ results in the equation, which contains only *n_l_*(*x*), and *H_l_*′(*x*), *H_l_*′″(*x*), *M*′(*x*). Solving this equation with respect to *n_l_*(*x*), we find:

$n_{l}\left(x\right)=-\frac{\left(1+\sigma\right)\left(4l_{l}^{2}+Ah_{l}^{2}\right)}{2\left(2+A\right)}H'''_{l}-\frac{2\left(h_{l}^{2}+A\left(h_{l}^{2}-l_{l}^{2}\right)\right)}{h_{l}^{2}\left(2+A\right)}H'_{l}+\frac{A\left(h_{l}^{2}-2l_{l}^{2}\right)}{h_{l}^{2}\left(2+A\right)}M'.$ (S10)

Substituting expressions (S6)-(S10) into the elastic energy functional (S3) and integrating over the neutral surfaces of the monolayers, we obtain the asymmetric bilayer deformation energy per unit length along the domain boundary.

**Elastic energy of symmetric bilayer**

For the symmetric bilayer, the elastic energy functional can be obtained from equation (S3) by substitution: *h_u_* = *h_l_* = *h*, *B_u_* = *B_l_* = *B*, *K_t_^u^* = *K_t_^l^* = *K_t_*, *K_a_^u^* = *K_a_^l^* = *K_a_*, *σ*_0_*^u^* = *σ*_0_*^l^* = *σ*_0_, *J_u_* = *J_l_* = *J*_0_:

$\begin{matrix} W_{S}=\int dx\left\{\frac{B}{2}\left(n'_{u}+J_{0}\right)^{2}+\frac{K_{t}}{2}\left(n_{u}-H'_{u}\right)^{2}+\frac{\sigma_{0}}{2}\left(H'_{u}\right)^{2}+\frac{K_{a}}{2h^{2}}\left(h-\frac{h^{2}}{2}n'_{u}+M-H_{u}-h\alpha_{0}\right)^{2}\right\} \\ +\int dx\left\{\frac{B}{2}\left(n'_{l}+J_{0}\right)^{2}+\frac{K_{t}}{2}\left(n_{l}+H'_{l}\right)^{2}+\frac{\sigma_{0}}{2}\left(H'_{l}\right)^{2}+\frac{K_{a}}{2h^{2}}\left(h-\frac{h^{2}}{2}n'_{l}-M+H_{l}-h\alpha_{0}\right)^{2}\right\}. \end{matrix}$ (S11)

The variation of the energy functional (S11) with respect to the functions *n_u_*, *n_l_*, *H_u_*, *H_l_*, *M* results in five Euler-Lagrange equations:

$\left\{\begin{matrix} \left(l^{2}+\frac{h^{2}A}{4}\right)n''_{u}-n_{u}-\frac{A}{2}M'+\left(1+\frac{A}{2}\right)H'_{u}=0, \\ \left(l^{2}+\frac{h^{2}A}{4}\right)n''_{l}-n_{l}+\frac{A}{2}M'-\left(1+\frac{A}{2}\right)H'_{l}=0, \\ \left(1+\frac{A}{2}\right)n'_{u}-\left(1+\sigma\right)H''_{u}+\frac{A}{h^{2}}\left(H_{u}-M-h\left(1-\alpha_{0}\right)\right)=0, \\ \left(1+\frac{A}{2}\right)n'_{l}+\left(1+\sigma\right)H''_{l}-\frac{A}{h^{2}}\left(H_{l}-M+h\left(1-\alpha_{0}\right)\right)=0, \\ n'_{u}-n'_{l}+\frac{2}{h^{2}}\left(H_{u}-M-h\right)+\frac{2}{h^{2}}\left(H_{l}-M+h\right)=0, \end{matrix}\right.$ (S12)

where *l* = (*B*/*K_t_*)^1/2^. Note, that the spontaneous curvature *J*_0_ does not enter the Euler-Lagrange equations. The general solution of the system (S12) can be written as:

$\begin{matrix} H_{u}\left(x\right)=h\left(1-\alpha_{0}\right)+c_{0}+c_{1}x+c_{2}e^{\sqrt{\frac{\sigma}{1+\sigma}}\frac{x}{l}}+c_{3}e^{-\sqrt{\frac{\sigma}{1+\sigma}}\frac{x}{l}}+c_{4}e^{q_{1}x}+c_{5}e^{q_{2}x}+c_{6}e^{-q_{1}x}+c_{7}e^{-q_{2}x}, \\ H_{l}\left(x\right)=-h\left(1-\alpha_{0}\right)+c_{0}+c_{1}x+c_{2}e^{\sqrt{\frac{\sigma}{1+\sigma}}\frac{x}{l}}+c_{3}e^{-\sqrt{\frac{\sigma}{1+\sigma}}\frac{x}{l}}-c_{4}e^{q_{1}x}-c_{5}e^{q_{2}x}-c_{6}e^{-q_{1}x}-c_{7}e^{-q_{2}x}, \\ M\left(x\right)=c_{0}+c_{1}x+\left(1+\frac{h^{2}\sigma}{2l^{2}}\right)\left(c_{2}e^{\sqrt{\frac{\sigma}{1+\sigma}}\frac{x}{l}}+c_{3}e^{-\sqrt{\frac{\sigma}{1+\sigma}}\frac{x}{l}}\right), \\ n_{u}\left(x\right)=c_{1}+\frac{\sqrt{\sigma\left(1+\sigma\right)}}{l}\left(c_{2}e^{\sqrt{\frac{\sigma}{1+\sigma}}\frac{x}{l}}-c_{3}e^{-\sqrt{\frac{\sigma}{1+\sigma}}\frac{x}{l}}\right)+ \\ +\frac{\left(c_{4}e^{q_{1}x}-c_{6}e^{-q_{1}x}\right)q_{1}\left(h^{2}\left(1+\sigma\right)\left(h^{2}A+4l^{2}\right)q_{1}^{2}+4h^{2}A+4h^{2}-4Al^{2}\right)}{2h^{2}\left(2+A\right)}+ \\ \frac{\left(c_{5}e^{q_{2}x}-c_{7}e^{-q_{2}x}\right)q_{2}\left(h^{2}\left(1+\sigma\right)\left(h^{2}A+4l^{2}\right)q_{2}^{2}+4h^{2}A+4h^{2}-4Al^{2}\right)}{2h^{2}\left(2+A\right)}, \\ n_{l}=n_{u}\left(x\right)-2c_{1}-2\frac{\sqrt{\sigma\left(1+\sigma\right)}}{l}\left(c_{2}e^{\sqrt{\frac{\sigma}{1+\sigma}}\frac{x}{l}}-c_{3}e^{-\sqrt{\frac{\sigma}{1+\sigma}}\frac{x}{l}}\right), \end{matrix}$ (S13)

where

$q_{1,2}=\sqrt{2}\sqrt{\frac{A\frac{l^{2}}{h^{2}}+\sigma-A\mp\sqrt{A^{2}\frac{l^{4}}{h^{4}}+\sigma^{2}\left(1+A\right)-\left(\sigma+\frac{2l^{2}}{h^{2}}\right)A\left(2+A+\sigma\right)}}{\left(1+\sigma\right)\left(h^{2}A+4l^{2}\right)}}$, (S14)

where *c*_0_, *c*_1_, *c*_2_, *c*_3_ are constant real coefficients, and *c*_5_, *c*_6_, *c*_7_, *c*_8_ are constant complex coefficients, which depend on the boundary conditions (4)–(7) appended by the requirement that all functions are real at any real *x*. The obtained expressions for the projections of directors *n_u_*(*x*) and *n_l_*(*x*), the shape of monolayer interface *M*(*x*) and neutral surfaces *H_u_*(*x*) and *H_l_*(*x*) allow finding the relative change of the area of the neutral surfaces *α_u_*(*x*) and *α_l_*(*x*) of the upper and lower monolayers using the condition of the local volumetric incompressibility (S2), which in the one-dimensional case looks as follows:

$\begin{matrix} H_{u}-M=h-\frac{h^{2}}{2}n'_{u}-h\alpha_{u}, \\ M-H_{l}=h-\frac{h^{2}}{2}n'_{l}-h\alpha_{l}. \end{matrix}$ (S15)

Substituting expressions (S13) into the elastic energy functional (S11) and integrating over the neutral surface of the monolayers, we obtain the symmetric bilayer deformation energy per unit length along the domain boundary.

**Elastic energy of monolayer**

The monolayer neutral surface does not exist in the location of an amphipathic peptide partially incorporated into the monolayer. Thus, the monolayer region opposing the peptide is in different conditions than the monolayer, which is a part of the lipid bilayer. For definiteness, we assume that the peptide is adsorbed to the upper monolayer of the membrane. For the monolayer region just beneath the peptide, we can write down the expression for the elastic energy in the following way:

$W_{M}=\frac{K_{t}}{2}\int dx \left(l^{2}\left(n'_{l}+J_{l}\right)^{2}+\left(n_{l}-N_{l}\right)^{2}+A\left(\alpha_{l}-\alpha_{0}\right)^{2}+\sigma\left(N_{l} \right)^{2}\right),$ (S16)

where *N_l_* is the projection of the unit normal of the lower monolayer neutral surface onto the *x*-axis. The variation of this energy functional with respect to the functions *n_l_*(*x*), *N_l_*(*x*), *α_l_*(*x*) yields the following equations:

$\left\{\begin{matrix} \alpha_{l}\left(x\right)=\alpha_{0}, \\ N_{l}\left(x\right)=\frac{n_{l}\left(x\right)}{1+\sigma}, \\ l^{2}n''_{l}-\frac{\sigma}{1+\sigma}n_{l}=0. \end{matrix}\right.$ (S17)

The general solution of the last equation is:

$n_{l}\left(x\right)=d_{1}e^{\sqrt{\frac{\sigma}{1+\sigma}}\frac{x}{l}}+d_{2}e^{-\sqrt{\frac{\sigma}{1+\sigma}}\frac{x}{l}},$ (S18)

where *d*_1_, *d*_2_ are real constant coefficients. The shape of the neutral surface of the monolayer is determined from the relation:

$N_{l}=\frac{n_{l}}{1+\sigma}=-H'_{l}$, (S19)

which yields:

$H_{l}\left(x\right)=d_{0}-d_{1}\frac{l}{\sqrt{\sigma\left(1+\sigma\right)}}e^{\sqrt{\frac{\sigma}{1+\sigma}}\frac{x}{l}}+d_{2}\frac{l}{\sqrt{\sigma\left(1+\sigma\right)}}e^{-\sqrt{\frac{\sigma}{1+\sigma}}\frac{x}{l}},$ (S20)

where *d*_0_ is the real constant coefficient. Now, the energy of the monolayer region (S16) can be written as:

$W_{M}=K_{t}l^{2}\left(d_{1}e^{\sqrt{\frac{\sigma}{1+\sigma}}\frac{x}{l}}+d_{2}e^{-\sqrt{\frac{\sigma}{1+\sigma}}\frac{x}{l}}\right)J_{l}+\frac{K_{t}}{2}l\sqrt{\frac{\sigma}{1+\sigma}}\left(d_{1}^{2}e^{2\sqrt{\frac{\sigma}{1+\sigma}}\frac{x}{l}}-d_{2}^{2}e^{-2\sqrt{\frac{\sigma}{1+\sigma}}\frac{x}{l}}\right).$ (S21)

We connect the monolayer of the adjacent bilayer and the monolayer opposing the peptide requiring the continuity of the directors *n_l_*(*x*) and the neutral surfaces *H_l_*(*x*) at the boundaries.
